# Diversity of *Trichoderma* species associated with green mold contaminating substrates of *Lentinula edodes* and their interaction

**DOI:** 10.3389/fmicb.2023.1288585

**Published:** 2024-01-08

**Authors:** Zi-Jian Cao, Juan Zhao, Yu Liu, Shou-Xian Wang, Su-Yue Zheng, Wen-Tao Qin

**Affiliations:** ^1^Institute of Plant Protection, Beijing Academy of Agriculture and Forestry Sciences, Beijing, China; ^2^School of Landscape and Ecological Engineering, Hebei University of Engineering, Handan, China

**Keywords:** Hypocreaceae, *Trichoderma*, green mold, *Lentinula edodes*, phylogeny, morphology, taxonomy

## Abstract

**Introduction:**

The contamination of *Trichoderma* species causing green mold in substrates poses a significant obstacle to the global production of *Lentinula edodes*, adversely impacting both yield and quality of fruiting bodies. However, the diversity of *Trichoderma* species in the contaminated substrates of *L. edodes* (CSL) in China is not clear. The purpose of this study was to assess the biodiversity of *Trichoderma* species in CSL, and their interactions with *L. edodes*.

**Methods:**

A comprehensive two-year investigation of the biodiversity of *Trichoderma* species in CSL was conducted with 150 samples collected from four provinces of China. *Trichoderma* strains were isolated and identified based on integrated studies of phenotypic and molecular data. Resistance of *L. edodes* to the dominant *Trichoderma* species was evaluated in dual culture *in vitro*.

**Results:**

A total of 90 isolates were obtained and identified as 14 different *Trichoderma* species, including six new species named as *Trichoderma caespitosus*, *T. macrochlamydospora*, *T. notatum*, *T. pingquanense*, *T. subvermifimicola*, and *T. tongzhouense*, among which, *T. atroviride*, *T. macrochlamydospora* and *T. subvermifimicola* were identified as dominant species in the CSL. Meanwhile, three known species, namely, *T. auriculariae*, *T. paraviridescens* and *T. subviride* were isolated from CSL for the first time in the world, and *T. paratroviride* was firstly reported to be associated with *L. edodes* in China. Notebly, the in vitro evaluation of *L. edodes* resistance to dominant *Trichoderma* species showed strains of *L. edodes* generally possess poor resistance to *Trichoderma* contamination with *L. edodes* strain SX8 relatively higher resistant.

**Discussion:**

This study systematically investigated the diversity of *Trichoderma* species in the contaminated substrate of *L. edodes*, and a total of 31 species so far have been reported, indicating that green mold contaminated substrates of edible fungi were undoubtedly a biodiversity hotspot of *Trichoderma* species. Results in this study will provide deeper insight into the genus *Trichoderma* and lay a strong foundation for scientific management of the *Trichoderma* contamination in *L. edodes* cultivation.

## 1 Introduction

*Lentinula edodes* (Berk.) Pegler is commonly known as “Xianggu” in Chinese, which is the most widely cultivated mushroom species in the world (Yan et al., 2020, 2021). The annual total production and sales rank first, and the total output of *L. edodes* in 2020 accounted for 29.25% of the total output of edible fungi in China (Zhang et al., 2015; Chinese Edible Fungus A, 2022). *Lentinula edodes* is distinguished by delicious taste, multiple nutrients, and medicinal properties (Zhao et al., 2020). The cultivation of *L. edodes* demonstrates inherent characteristics of circularity, high efficiency and ecological compatibility, rendering it a prime avenue for farmers to alleviate poverty and facilitate rural revitalization in China (He, 2019). However, the substrate contamination from *Trichoderma* spp. is regarded as a significant constraint in the production of *L. edodes*, resulting in economic losses of 10–20% of the total production (Yan et al., 2019). The investigation into the deleterious effects of *Trichoderma* spp. on edible fungi commenced relatively late in China. Following the rapid development of edible fungi industry, serious *Trichoderma* spp. contamination reported after the 1980s, with the most major mold contamination occurred in summer, resulting in entire cultivation batches being discarded (Fu et al., 1981). “Green mold on *L. edodes* and its chemical control” was formally reported at the National Fungal Lichen Academic Symposium in 1990; then, *Trichoderma* contamination was officially listed as a disease of *L. edodes* recorded in history (Wang, 1994). Meanwhile, there is no highly effective fungicide against *Trichoderma*, which is harmless to *L. edodes*. The identification and detection of *Trichoderma* species are essential for the prevention and control of green mold contamination in the production of *L. edodes* (Lee et al., 2020; Allaga et al., 2021).

The genus *Trichoderma* Pers. (Ascomycota, Sordariomycetes, Hypocreales) is cosmopolitan and of great diversity with over 441 known species until now (Barrera et al., 2021; Zheng et al., 2021; Cao et al., 2022; Yu et al., 2022). Among them, many species, such as *T. atroviride*, *T. harzianum*, and *T. pleuroticola*, are contaminants associated with the production of edible fungi (Kim et al., 2012). However, the invasiveness and isolation frequencies of *Trichoderma* spp. are distinct on edible fungi in different regions (Kim et al., 2012; Wang et al., 2016). Furthermore, the recognition of *Trichoderma* spp. associated with the contamination of edible fungi primarily was previously conducted only by morphological characteristic and ITS sequence analysis. In addition, the samples collected were limit to local regions, which are difficult for systematic understanding of their population structure. There is also a lack of systematic research on the lineage of *Trichoderma* spp. contaminating on *L. edodes* in multiple regions (Jiang et al., 1995; Hatvani et al., 2007; Cai and Druzhinina, 2021). Considering the constraints faced by the edible fungi industry, a significant long-term objective for *L. edodes* breeding is the selection of varieties with superior resistance to *Trichoderma* contamination under the interaction between *Trichoderma* species and *L. edodes*.

To elucidate the *Trichoderma* spp. present in the CSL in North China, we conducted an in-depth investigation on the microbial community within CSL and identified six novel species belonging to the Harzianum clade, which have not been previously described. Their phylogenetic positions were determined based on sequence analyses of partial translation elongation factor 1-alpha (*tef1-α*) and partial RNA polymerase second largest subunit (*rpb2*) genes. Similarities and differences of morphological characters between the new species and their closely related species were presented and compared in detail. Their interaction was also primarily discussed in this study to distinguish the resistance difference of mushroom varieties to *Trichoderma*.

## 2 Materials and methods

### 2.1 Isolates and specimens

**Figure 1:**
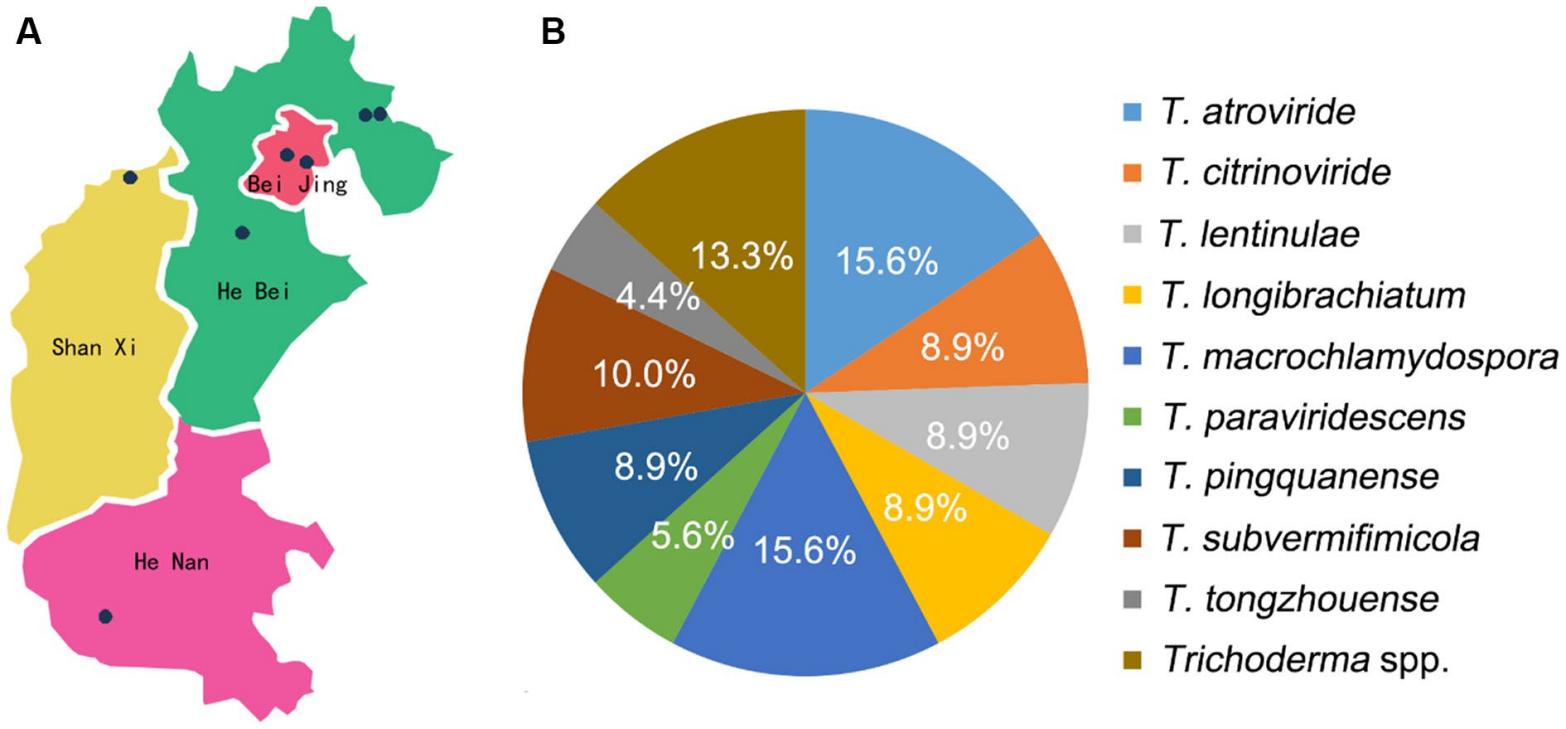
Isolation and identification of *Trichoderma* spp. **(A)** Map of isolates collection; **(B)** isolation frequency of *Trichoderma* spp.

Cylindrical sticks wrapped in plastic bags are usually used for *L. edodes* cultivation in the main production areas investigated. Contaminated stick specimens of *L. edodes* by *Trichoderma* spp. were separately collected from several edible fungus production bases in seven regions of Beijing, Hebei, Shanxi, and Henan Provinces of China during 2020 to 2022 (Figure 1A). To make the isolation frequency more convincing, contaminated *L. edodes* sticks were randomly collected. These sticks were kept as intact as possible to avoid secondary contamination due to breakage. Cultures were obtained by aseptically transferring the contaminated substrates of collected stick specimens onto potato dextrose agar (PDA) following the method of Kim et al. (2010) (Supplementary Figure S1). The isolation frequency (%) for *Trichoderma* species was calculated: the number of isolates per species/total number isolates × 100.

Colonies with features resembling typical *Trichoderma* spp. were preliminary screened and then purified with monoconidial isolation method, and single-conidium cultures were stored in 25 vol % glycerol at −80°C (Chomnunti et al., 2014). The strains obtained were deposited in the culture collection of the Beijing Academy of Agricultural and Forestry Sciences, China.

### 2.2 Morphology and growth characterization

For morphological studies, growth rates and colony characteristics were determined on three different media: potato dextrose agar (PDA; 200 g potato, 18 g dextrose, 18 g agar, 1 L distilled water), cornmeal dextrose agar (CMD; 40 g cornmeal, 20 g glucose, 18 g agar, 1 L distilled water), and synthetic low nutrient agar (SNA; 1 g KH_2_PO_4_, 1 g KNO_3_, 0.5 g MgSO_4_‧7H_2_O, 0.5 g KCl, 0.2 g glucose, 0.2 g sucrose, 18 g agar, 1 L distilled water), and cultured at 25, 30, and 35°C in darkness, respectively. Mycelial disks (5 mm diam.) were incubated in Petri dishes (9 cm diam.) with each isolate three replicates. Colony radius was measured after 72 h. The morphological characteristics of colonies, including colony appearance, color, radial, concentric rings formed by spore production, pigmentation, and odor, were recorded (Zheng et al., 2021). Microscopic characters were photographed and measured with Olympus BX51 microscope (Tokyo, Japan) connected to a DP controller digital camera. Microscopic characteristics and micromorphological data were examined on the cultures grown on SNA and PDA for 7 days at 25°C. Descriptions included the data of phialides, conidia, and chlamydospores. New species were described basically following counterparts by Chaverri et al. (2015).

### 2.3 DNA extraction, PCR amplification, and sequencing

Mycelium of each isolate was cultivated on PDA medium for 7 days at 25°C in darkness; then, genomic DNA was extracted using the Plant Genomic DNA Kit (DP305, TIANGEN Biotech, Beijing, China). The DNA fragments of *tef1-α* and *rpb2* were amplified and sequenced with the primer pairs EF1-728F (Carbone and Kohn, 1999) and TEF1LLErev (Jaklitsch et al., 2005), and fRPB2-5f and fRPB2-7cr (Liu et al., 1999), respectively. Polymerase chain reactions (PCRs) were performed in 25 μL total volumes consisting of 12.5 μL Premix Taq™ (TaKaRa Taq™ Version 2.0 plus dye), 1.0 μL of each primer (10 μM), 1.5 μL DNA, and 9 μL double sterilized water. Cycling parameters were 95°C for 5 min, followed by 30 cycles of 95°C for 1 min, 55°C for 1 min, and 72°C for 2 min, with a final elongation at 72°C for 10 min. The positive amplicons were sequenced by ABI 3730 DNA Sequencer (Applied Biosystems, Bedford, MA, USA) at SinoGenoMax company.

### 2.4 Phylogenetic analyses

All sequences were assembled and manually adjusted using the DNAStar Seqman program 7.1.0 (DNASTAR. Inc., Madison). Phylogenetic analyses were performed for the identification based on the combined sequences of *rpb2* and *tef1-α*. Sequences generated from this study and those retrieved from GenBank based on previous studies are listed in Supplementary Table S1. The final dataset for analyses comprised 289 sequences representing 149 *Trichoderma* species, with *Nectria berolinensis* and *N. eustromatica* selected as outgroup taxa. Alignments were performed separately for each molecular marker and converted to nexus files with Clustal X 1.83 (Thompson et al., 1997). Then, the *rpb2* and *tef1-α* alignments were manually adjusted and concatenated through BioEdit v.7.0. Ambiguously aligned regions were excluded from phylogenetic analyses. Phylogenetic analyses were performed using both maximum parsimony (MP) and Bayesian tree inference (BI).

Maximum parsimony tree was performed using PAUP 4.0b10. Each iteration performs 1,000 iterations of random sequence addition by heuristic search and subsequent branch-swapping algorithm using tree-bisection-reconnection (TBR) (Swofford, 2002). The topological confidence of the tree was tested by calculating maximum parsimony bootstrap proportion (MPBP). Bootstrap analyses calculated via 1,000 bootstrap replications, each with 10 replicates of random addition of taxa.

Bayesian inference trees were analyzed using MrBayes v. 3.1.2 (Ronquist and Huelsenbeck, 2003). GTR + I + G were estimated as the best-fit model using MrModeltest 2.3 (Nylander, 2004). Metropolis-coupled Markov chain Monte Carlo (MCMCMC) analyses were run using four chains for 6,000,000 generations sampling every 100 generations. The initial 25% tree sample was discarded as burn-in, and Bayesian inference posterior probability (BIPP) was determined from the remaining trees. Trees were visualized in FigTree v1.4.3 (Zhang and Zhuang, 2019).

### 2.5 Resistance evaluation of *Lentinula edodes* to *Trichoderma* in dual culture

To evaluate the resistance of *L. edodes* to two dominant new *Trichoderma* species, *T. macrochlamydospora* and *T. subvermifimicola*, 35 strains of *L. edodes* were selected for dual-culture experiments on PDA (Supplementary Table S2). *Trichoderma* and *L. edodes* strains were first cultured on PDA for 5 and 10 days, respectively; then, mycelial disks (5 mm diameter) of *L. edodes* were inoculated onto PDA one side of the Petri plates (9 cm diameter). Six days later, *Trichoderma* mycelial disks of equal size were placed on a fresh PDA plate 50 mm apart from *L. edodes*. As control, *L. edodes* alone were placed in Petri dishes without *Trichoderma*. The plates were incubated in darkness at 25°C. Each assay was repeated three times.

Microscopic features of the hyphal interaction were photographed by DP controller digital camera attached to an Olympus BX51 microscope (Tokyo, Japan) after colonies of *Trichoderma* and *L. edodes* contacted with each other in 24–48 h. Status of cultures including growth rate and sporulation of *Trichoderma* spp. on *L. edodes* were photographed at the 13th day after inoculation. The inhibitory rate (%) was used to characterize the resistance of *L. edodes* to *Trichoderma* spp. as (colony radius of *L. edodes* in control group – colony radius in treatment group) / (colony radius of *L. edodes* in control group – 2.5 mm) × 100.

## 3 Results

### 3.1 Isolation and identification of *Trichoderma* spp.

In this study, 90 isolates of *L. edodes* contaminated with *Trichoderma* spp. were collected from seven regions of Beijing, Hebei, Shanxi, and Henan provinces during 2020 to 2022. These isolates were then recognized as 14 species, including eight species in the Harzianum clade, two species in the Longibrachiatum clade, and four species in the Viride clade. Twelve strains collected from Beijing belonged to four species, among which *T. caespitosus*, *T. notatum*, and *T. tongzhouense* were new species. The 71 strains from Hebei Province represented 11 species, including three new species as *T. macrochlamydospora*, *T. pingquanense*, and *T. subvermifimicola*. In addition, the isolates collected from Henan province were recognized as three species, and four strains collected from Shanxi province were all recognized as *T. macrochlamydospora*. The isolation frequency of 14 *Trichoderma* species was calculated and analyzed. Among them, isolation frequencies of *T. atroviride*, *T. macrochlamydospora*, and *T. subvermifimicola* rank the top three, namely, 15.6, 15.6, and 10.0%, respectively (Figure 1). Three known *Trichoderma* species, namely, *T. auriculariae*, *T. paraviridescens* and *T. subviride* were isolated from CSL for the first time in the world, and *T. paratroviride* was firstly reported to be associated with *L. edodes* in China. The information of the specimens used in this study is shown in Figure 1 and Supplementary Table S3. Interestingly, each stick was usually contaminated by single *Trichoderma* species and occasionally two *Trichoderma* species.

### 3.2 Phylogenetic analyses

**Figure 2:**
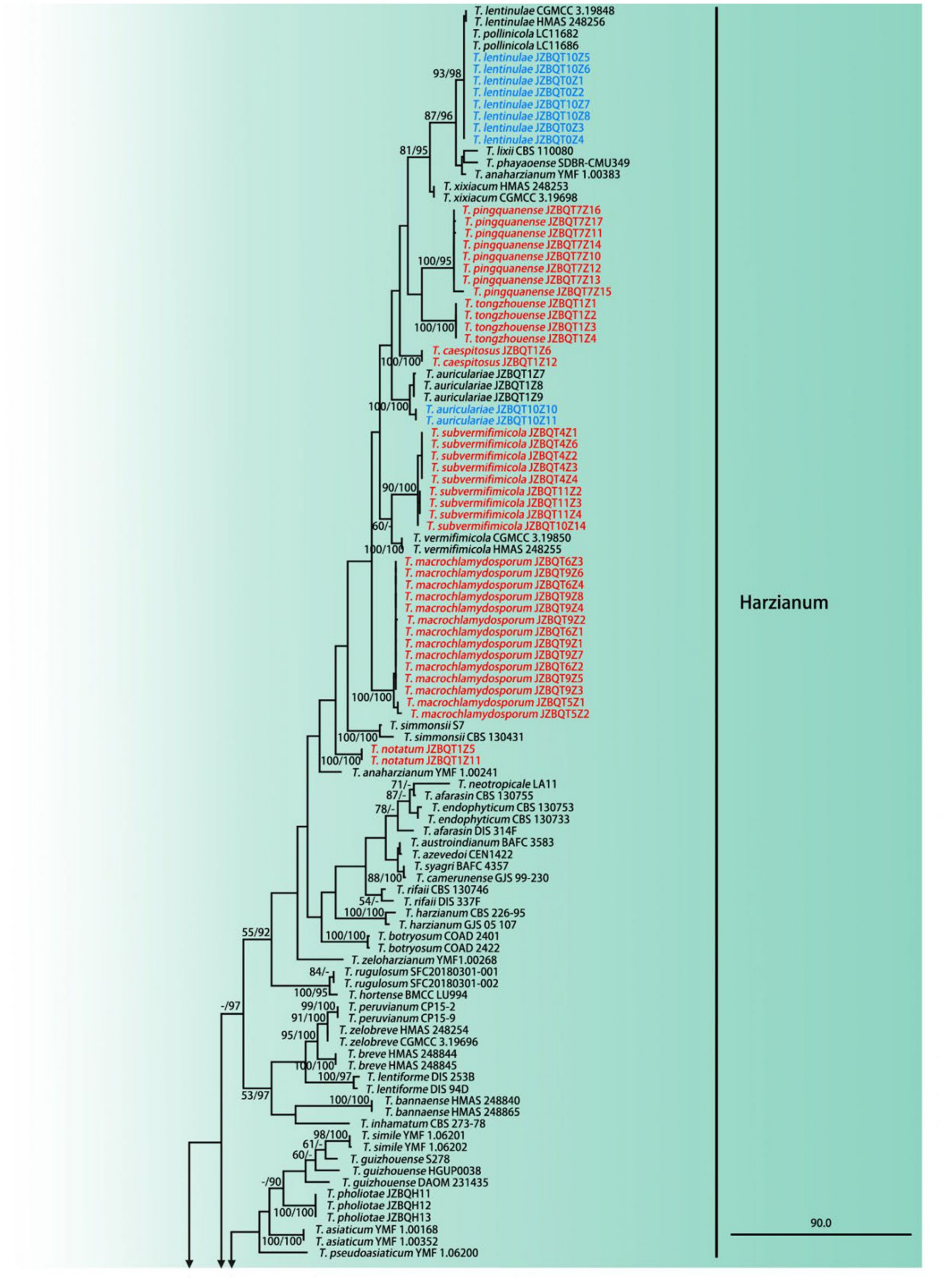

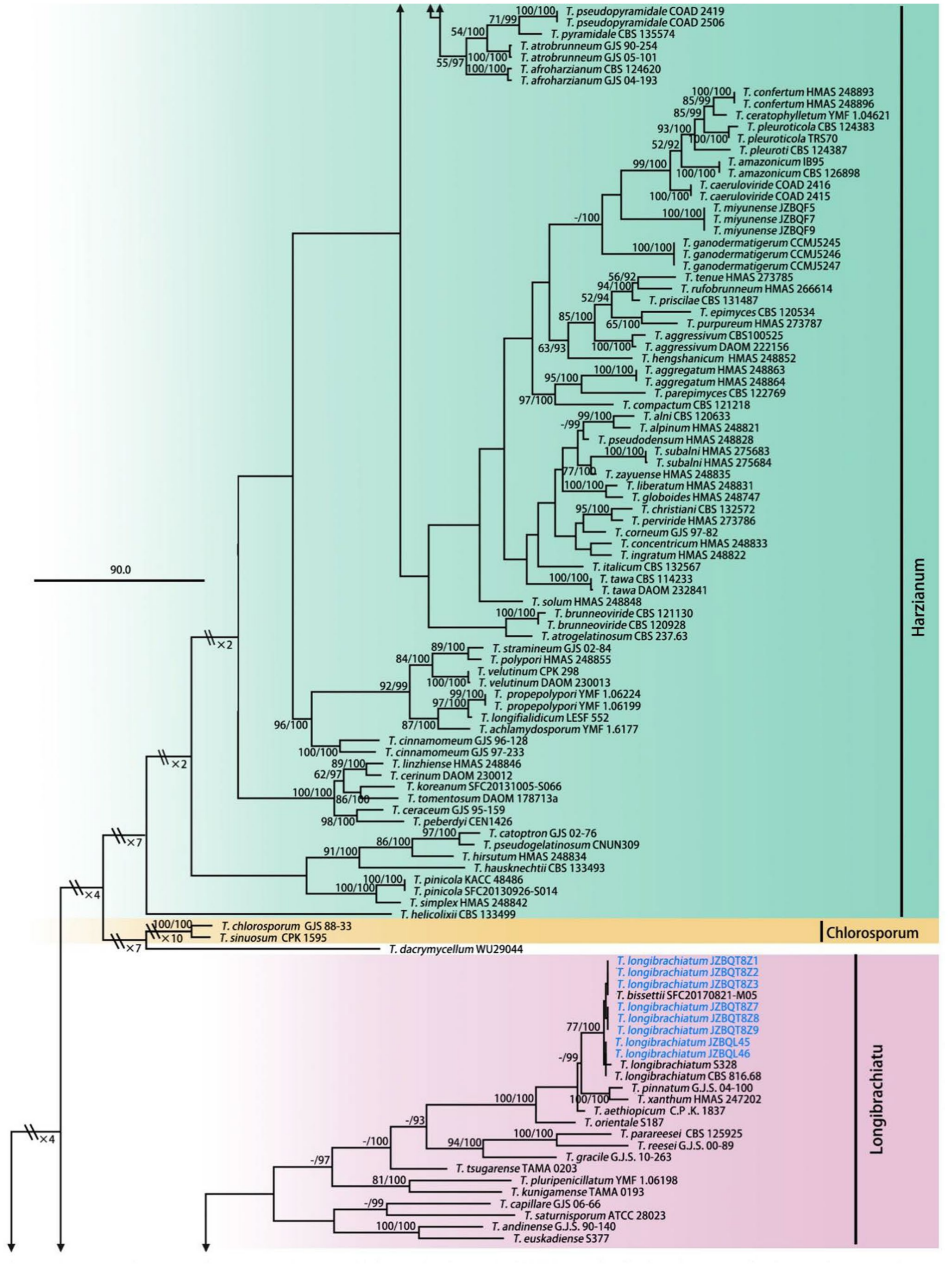

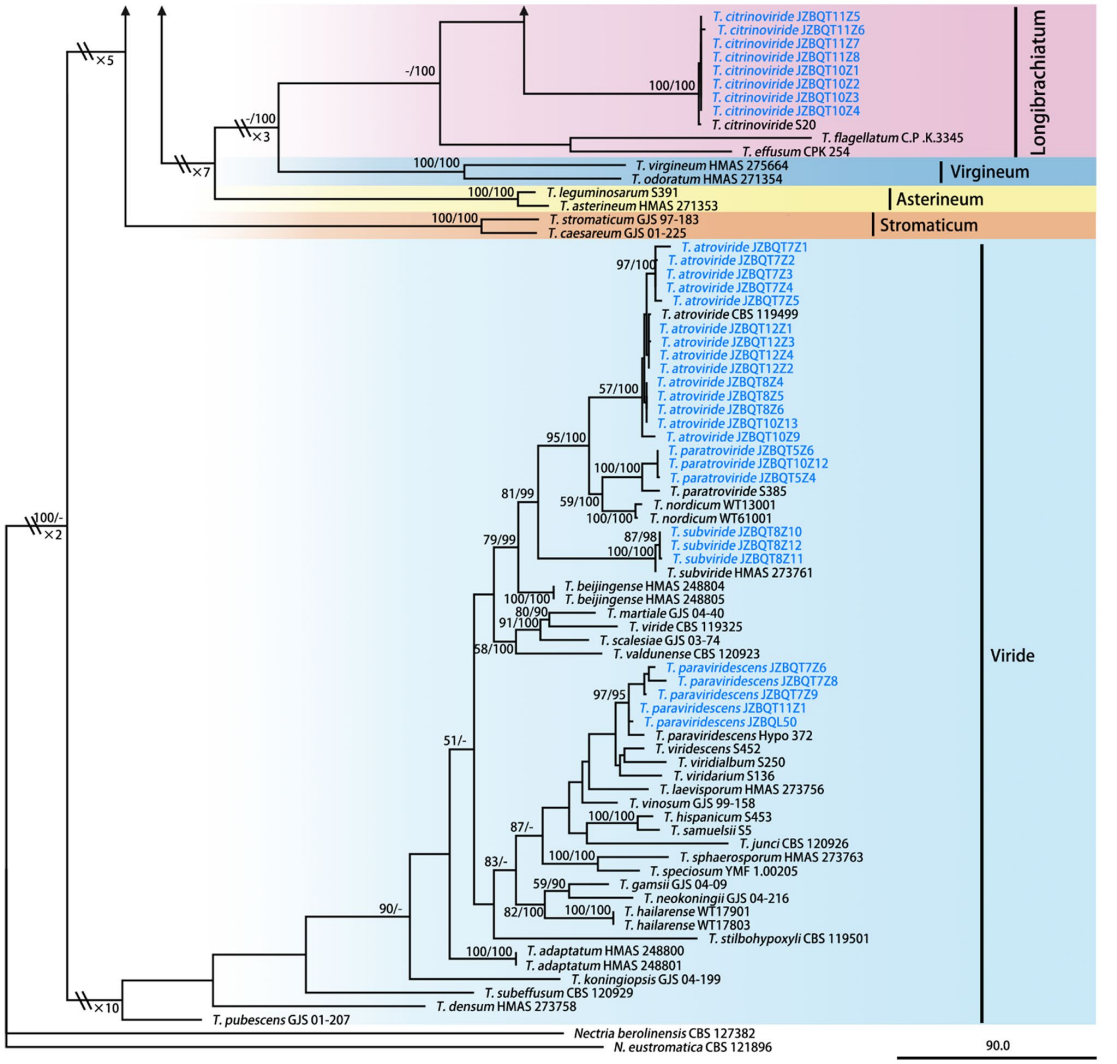
Maximum parsimony phylogram of investigated *Trichoderma* species inferred from the combined sequences of *rpb2* and *tef1-α*. MPBP above 50% **(left)** and BIPP above 90% **(right)** were indicated at the nodes. New species proposed are indicated in red font, and known species in this study were indicated in blue font. Shorted nodes are marked with crossing lines and indications (×2, ×3, ×4, ×7, ×10) of how many times the node has been shortened.

The partition homogeneity test (*p* = 0.01) of *rpb2* and *tef1-α* sequences indicated that the individual partitions were generally congruent (Cunningham, 1997). Phylogenetic positions of *Trichoderma* species were determined by analyses of the combined *rpb2* and *tef1-α* dataset containing 289 taxa and 2,440 characters, of which 1,184 characters were constant, 275 variable characters were parsimony-uninformative, and 981 were parsimony-informative. Two most-parsimonious trees with the same topology were generated (Figure 2) (tree length = 7,876, CI = 0.2920, HI = 0.7080, RC = 0.2469, and RI = 0.8455). Meanwhile, the BI analysis showed congruence with the topology of the MP analyses.

A total of 289 sequences representing 149 *Trichoderma* species, including our seven new species and two out-groups, were used to construct the phylogenetic tree. All species formed a strongly supported group (MPBP = 100%), which was generally congruent with the previous studies (Gu et al., 2020).

Phylogenetic analyses revealed that six new species belonged to the Harzianum clade and clustered at the top of the tree. Their phylogenetic relationships were relatively close. *Trichoderma tongzhouense* and *T*. *pingquanense*, and *T. subvermifimicola* and *T. vermifimicola* (MPBP = 60%) clustered as two sister groups, respectively. *Trichoderma macrochlamydospora*, *T. caespitosus*, and *T*. *notatum* formed highly supported terminal branches with statistic values MPBP/BIPP = 100%/100%. In addition, *T. auriculariae* and *T. lentinulae* also belonged to the Harzianum clade. Other known species, *namely, T. longibrachiatum*, *T. paraviridescens*, *T. paratroviride*, *T. atroviride*, *T. subviride*, and *T. citrinoviride*, isolated in this study belonged to the Longibrachiatum and Viride clade. Their phylogenetic positions were consistent with previous studies (Gu et al., 2020). Meanwhile, high degree of intraspecies variation for *T. paratroviride* and *T. paraviridescens* was accepted.

### 3.3 Taxonomy

***Trichoderma atroviride*** P. Karst., Bidr. Känn. Finl. Nat. Folk 51: 363. 1892. Wen, Tao & Chen, Acta Mycol. Sin. 12: 124. 1993.

= *Hypocrea atroviridis* Dodd, Lieckf. & Samuels, Mycologia 95: 36. 2003.

*Materials examined*: China, Hebei Province, Pingquan, 1 January 2022, W.T. Qin, X.Q. Wang, JZBQT7Z1, JZBQT7Z2, JZBQT7Z3; *ibid.*, 8 January 2022, W.T. Qin, X.Q. Wang, JZBQT8Z4, JZBQT8Z5, JZBQT8Z6; *ibid.*, 11 February. 2022, W.T. Qin, X.Q. Wang, JZBQT10Z9, JZBQT10Z13; *ibid.*, 8 Mar. 2022, W.T. Qin, X.Q. Wang, JZBQT12Z1, JZBQT12Z2, JZBQT12Z3.

Notes—*Trichoderma atroviride* is a widely distributed species found in many countries and is critical biological control agents promoting efficient plant growth and improving stress resistance (Skoneczny et al., 2015). In addition, green mold caused by *T*. *atroviride* was found to be associated with various edible fungi around the word, such as *L. edodes*, *Pleurotus* spp., *Agaricus* spp., and *Ganoderma lingzhi* (Kim et al., 2012; Yan et al., 2019). Its main characteristic is the smell of coconut, and subglobose, dark green conidia. Phialides slender, narrow ampulliform, often arcuate toward the end of the branches.

***Trichoderma auriculariae*** Z. J. Cao & W.T. Qin. J. Fungi 8: 7. 2022.

*Materials examined*: China, Hebei Province, Pingquan, 11 February 2022, W.T. Qin, X.Q. Wang, JZBQT10Z10, JZBQT10Z11.

*Notes*: *Trichoderma auriculariae* was originally found in the contaminated substrate of *Auricularia auricular* (Cao et al., 2022). It was isolated from the CSL for the first time. *Trichoderma auriculariae* has a typical conical branch of the Harzianum clade, with distinct symmetrical branches on both sides of the principal axis, often with 3–5 phialides whorled at the end of the branch. Notably, *T. auriculariae* had longer phialides than *T. simmonsi* and had larger conidia than that of *T. vermifimicola* and *T. xixiacum*. Interestingly, characteristics of *T. auriculariae* varied in its original host isolated from. In this study, strain JZBQT10Z10 isolated from the contaminated substrates of *L. edodes* grew faster [27–30 mm] and had stronger tolerance to high temperature than that of strain JZBQT1Z7 isolated from the contaminated substrate of *A. auricular* [5–7 mm] at 35°C (Cao et al., 2022).

**Figure 3:**
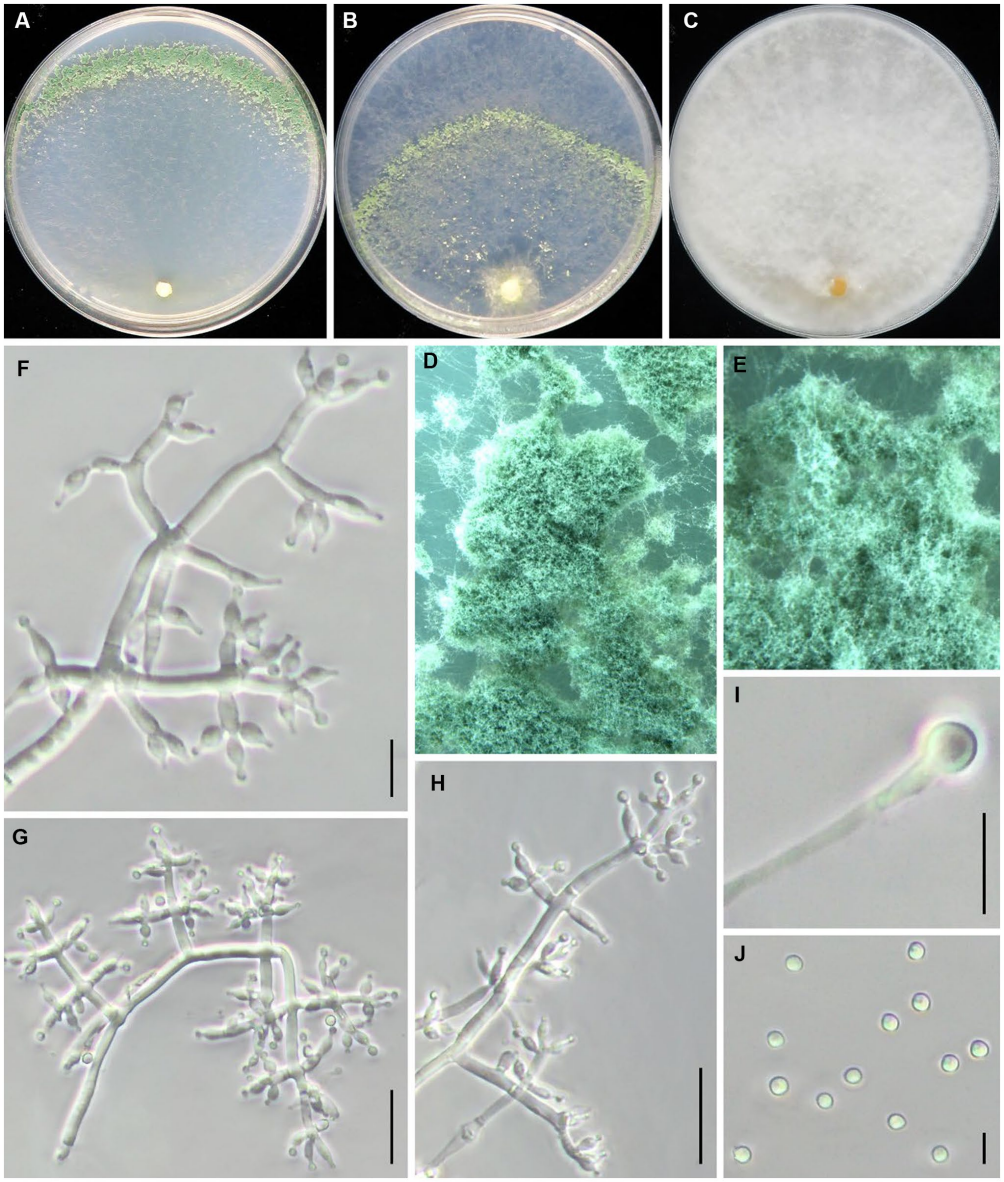
*Trichoderma caespitosus* (JZBQT1Z6) cultures at 25°C after 7 days [**(A)** on CMD, **(B)** on SNA, **(C)** on PDA]; **(D,E)** conidiation pustules (CMD, 7d); **(F–H)** conidiophores and phialides (SNA, 7d); **(I)** chlamydospores (PDA, 7 days); **(J)** conidia (SNA, 7 days); scale bars: **(F,G,H,I)** = 10 μm, **(J)** = 5 μm.

***Trichoderma caespitosus*** Z. J. Cao & W.T. Qin, sp. nov. (Figure 3).

MycoBank: MB849036.

*Type:* China, Beijing, Tongzhou district, 39°41′51” N, 116°45′1″ E, 26 August 2021, W.T. Qin, Y. Liu, S.X. Wang, (ex-type strain JZBQT1Z6). GenBank accessions: *rpb*2 = OP832383, *tef1-α* = OP832398.

*Etymology*: The specific epithet refers to dense conidiophores of this species.

*Description*. On CMD after 72 h, colony radius 65–68 mm at 25°C, covering the plate at 30°C, 22–25 mm at 35°C. Colony is round and hyaline, with very few airborne mycelia. A large number of white pustules formed obvious concentric rings along the outer edge of colony after 2 days. Conidiation formed on aerial hyphae and pustules and gradually turn pale green. No diffusing pigment. On PDA after 72 h, colony radius 60–63 mm at 25°C, 63–67 mm at 30°C, 8–10 mm at 35°C. Colony radial, dense, regularly round. Colony is white at 25°C and green at 30°C. Aerial hyphae white and abundant, denser at the colony center. Conidiation effuse in aerial hyphae. No diffusing pigment, no distinct odor. On SNA after 72 h, colony radius 48–52 mm at 25°C, 40–45 mm at 30°C, 12–13 mm at 35°C. Colony hyaline, aerial hyphae lacking. There were more aerial hyphae near the inoculation block, and pustules were formed in the middle circle, which gradually turned light green after 3 days. No diffusing pigment.

Conidiophores pyramidal, with a relatively obvious main axis, often rebranching 1–3 times. The branches are usually at right angles to the main axis, and phialides paired or in whorls of 3–4, rarely solitary at the end of the branches. Phialides ampulliform to lageniform, 4.8–9.9 × 2.1–3.4(−3.7) μm, l/w 1.4–3.1(−3.9), (1.1–)1.3–2.3 μm wide at the base (*n* = 45). Conidia green, smooth, globose to subglobose, 2.7–3.3 × 2.5–3.3 μm, l/w 1.0–1.2 (*n* = 30). Chlamydospores common, intercalary or terminal, variable in shape, ellipsoid, globose or oblong, 4.8–8.4 × 4.0–7.7 μm (*n* = 30).

*Additional strains examined*: China, Beijing, Tongzhou district, 39°41′51” N, 116°45′1″ E, 26 August 2021, W.T. Qin, Z.J. Cao, JZBQT1Z12.

*Notes*: The two strains of *T. caespitosus* formed a separate lineage in relation to *T. tongzhouense* and *T. pingquanense*. Their morphology was similar to each other, and their *tef1–α* sequences were nearly the same. *Trichoderma caespitosus* possessed 33 bp sequence divergences among 1,061 bp for *rpb2* (96.89%) from *T. tonzhouense* (strain JZBQT1Z1) and *T. pingquanense* (strain JZBQT7Z10). In addition, *T. caespitosus* form dense conidiophores pustules on CMD and SNA.

***Trichoderma citrinoviride*** Bissett, Can. J. Bot. 62: 926. 1984.

*Materials examined*: China, Hebei Province, Pingquan, 11 February. 2022, W.T. Qin, X.Q. Wang, JZBQT10Z1, JZBQT10Z2, JZBQT10Z3, JZBQT10Z4; *ibid.*, 3 March 2022, W.T. Qin, X.Q. Wang, JZBQT11Z5, JZBQT11Z6, JZBQT11Z7, JZBQT11Z8.

*Notes*: *Trichoderma citrinoviride* belongs to the clade of Longibrachiatum, which has been reported to be associated with *L. edodes* and *P*. *ostreatus*. It grows in a wide range of temperature. The asexual morphology of *T. citrinoviride* is similar to that of *T. atroviride*. According to previous study, the invasion ability of *T. citrinoviride* to *L. edodes* was weaker than that of *T. atroviride* (Park et al., 2005; Kim et al., 2012). This study shows that it is a prevalent species in the CSL in northern China.

***Trichoderma lentinulae*** J. Z. Sun & X.Z. Liu, MycoKeys 73: 116. 2020.

*Materials examined*: China, Beijing, Haidian district, 3 August 2021, W.T. Qin, Q. Gao, Z. J. Cao, JZBQT0Z1, JZBQT0Z2, JZBQT0Z3, JZBQT0Z4; China, Hebei Province, Pingquan, 11 February. 2022, W.T. Qin, X.Q. Wang, JZBQT10Z5, JZBQT10Z6, JZBQT10Z7, JZBQT10Z8.

*Notes*: *Trichoderma lentinulae* is first reported in contaminated fruiting bodies, strains, and plant rhizosphere soils, with shorter phialides than close-related species (Gu et al., 2020). *Trichoderma lentinulae* is the dominant species contamination in the production of *L. edodes* in Guizhou, China, and its metabolites and volatile products had obvious inhibitory effects on the mycelial growth of *L. edodes* (Wang, 2021). In this study, the isolation frequency of *T. lentinulae* was 8.9%, which was a potential threat to the production of *L. edodes* in northern China.

***Trichoderma longibrachiatum*** Rifai, Mycol. Pap. 116: 42. 1969.

*Materials examined*: China, Henan Province, Nanyang, 20 April 2021, W.T. Qin, JZBQL50; China, Hebei Province, Pingquan, 8 January. 2022, W.T. Qin, X.Q. Wang, JZBQT8Z1, JZBQT8Z2, JZBQT8Z3, JZBQT8Z7, JZBQT8Z8, JZBQT8Z9.

*Notes*: *Trichoderma longibrachiatum* is a species with wide range of sources, which can be found in soil and marine (Jaklitsch and Voglmayr, 2015; Kim et al., 2020). Studies have shown that *T. longibrachiatum* is also a common competitor of *L. edodes* with less invasive to *L. edodes* than *T. harzianum.* In addition, the growth rate patterns of *T. longibrachiatum* are a wide range at optimal temperatures (Kim et al., 2012). Pustules formed concentric circles, and *T. longibrachiatum* diffused yellow pigment, sometimes with white spots.

**Figure 4:**
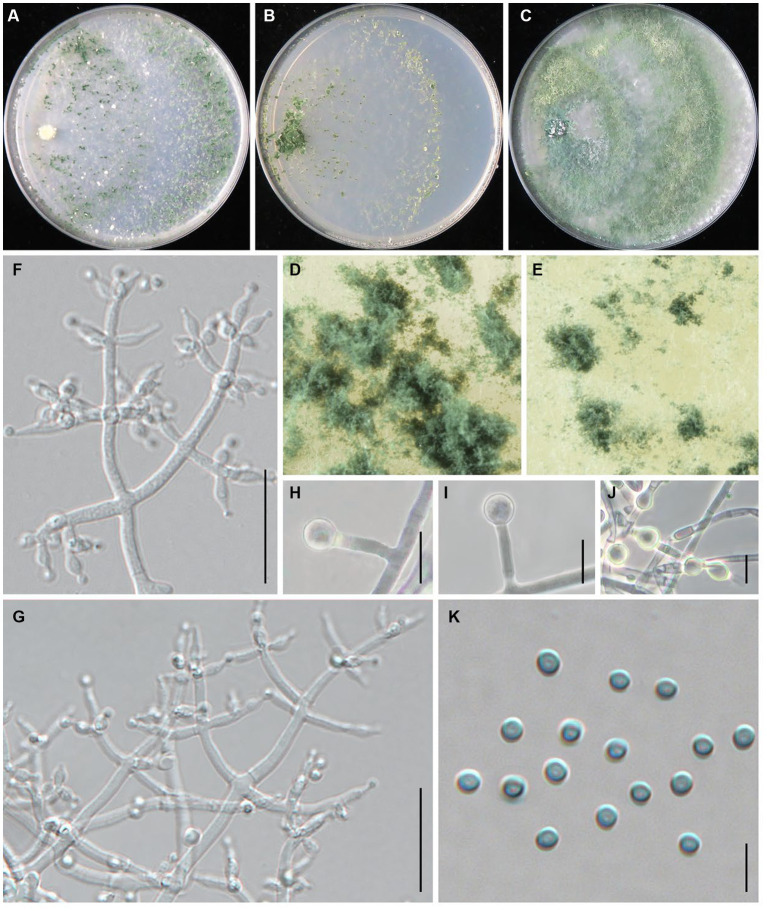
*Trichoderma macrochlamydospora* (JZBQT5Z1) cultures at 25°C after 7 days [**(A)** on CMD, **(B)** on SNA, **(C)** on PDA]; **(D,E)** conidiation pustules (CMD, 7 days); **(F,G)** conidiophores, phialides, and conidia (SNA, 7 days); **(H–J)** chlamydospores (PDA, 7 days); **(K)** conidia (SNA, 7 days); scale bars: **(F,G)** = 20 μm, **(H–J)** = 10 μm, **(K)** = 5 μm.

***Trichoderma macrochlamydospora*** Z. J. Cao & W.T. Qin, sp. nov. (Figure 4).

MycoBank: MB849037.

*Type*: China, Shanxi Province, Datong, Guangling, 39°47′22” N, 114°18′8″ E, 3 December 2021, Y. Liu, W.T. Qin. (ex-type strain JZBQT5Z1). GenBank accessions: ITS = ON653399, *rpb*2 = ON649955, *tef1-α* = ON649902.

*Etymology*: The specific epithet refers to the large chlamydospore.

*Description*: On CMD after 72 h, colony radius 67–70 mm at 25°C, 73–74 mm at 30°C, 13–15 mm at 35°C. Colony hyaline, indistinctly zonate, mycelium loose. Aerial hyphae short, inconspicuous. Many white conidia formed after 3 days of incubation, scant, gradually turning blue-green. Pustules abundant, spreading throughout the colony. No diffusing pigment, no distinct odor. On PDA after 72 h, colony radius 58–62 mm at 25°C, 58–66 mm at 30°C, 8–12 mm at 35°C. Colony white-green to green, distinctly zonate, mycelium dense and radial. Aerial hyphae long, abundant, spreading throughout the colony, forming a loose, floccose mat. Conidiation effuse in the aerial hyphae. No diffusing pigment, odor fruity. On SNA after 72 h, colony radius 54–55 mm at 25°C, 55–58 mm at 30°C, 8–10 mm at 35°C. Colony hyaline, circular, distinctly zonate; mycelia loose. Very small amounts of conidia were observed after 3 days of incubation, starting around the inoculum, first white, turning green. No diffusing pigment, no distinct odor.

Conidiophores pyramidal, with a relatively obvious main axis, often rebranching 1–3 times, side branches paired, more or less symmetrical. Phialides typically formed in whorls of 3–4, rarely solitary or paired, variable in shape and size, ampulliform to lageniform, (4.1–)4.7–11 × (2.0–)2.3–3.8 μm, l/w 1.4–5.5(−6.0), 1.2–2.1 μm wide at the base (*n* = 50). Conidia green, smooth, globose, 2.5–3.4 × 2.5–3.2 μm, l/w 1.0–1.1 (*n* = 50). Chlamydospores common, intercalary or terminal, variable in shape, ellipsoid, globose or oblong, 5.0–7.8(−10.8) × 4.7–7.9 μm (*n* = 25).

*Additional strains examined*: China, Shanxi Province, Datong, Guangling, 39°47′22” N, 114°18′8″ E, 3 Dec.2021, Y. Liu, W.T. Qin, JZBQT5Z2; China, Hebei Province, Chengde, Fengning, 41°14′49” N, 117°3′34″ E, 19 Dec. 2021, S.X. Wang, W.T. Qin, JZBQT6Z1, JZBQT6Z2, JZBQT6Z3, JZBQT6Z4; *ibid.*, 12 Jan. 2022, S.X. Wang, W.T. Qin, JZBQT9Z1, JZBQT9Z2, JZBQT9Z3, JZBQT9Z4.

*Notes*: In this study, *T. macrochlamydospora* is a common species with the isolation frequency 15.6% (14/90), indicating a potential threat to the production of *L. edodes* in the area investigated. *Trichoderma macrochlamydospora* shares a common ancestor with *T. subvermifimicola*, *T. vermifimicola*, and *T. simmonsii*, and they were similar in conidiophores, similar size and shape of phialides and conidia. *Trichoderma macrochlamydospora* is most similar to *T. subvermifimicola*, except larger chlamydospores (4.0–6.9 × 3.5–6.4 μm). As far as sequence divergences are concerned, 27 bp and 30 bp differences among 1,117 bp (97.58%) and 1,117 bp (97.31%) for *rpb2* were detected between *T. macrochlamydospora* (strain JZBQT5Z1) and its closely related species *T. subvermifimicola* (strain JZBQT4Z1) and *T. vermifimicola* (strain HMAS 248255), respectively.

**Figure 5:**
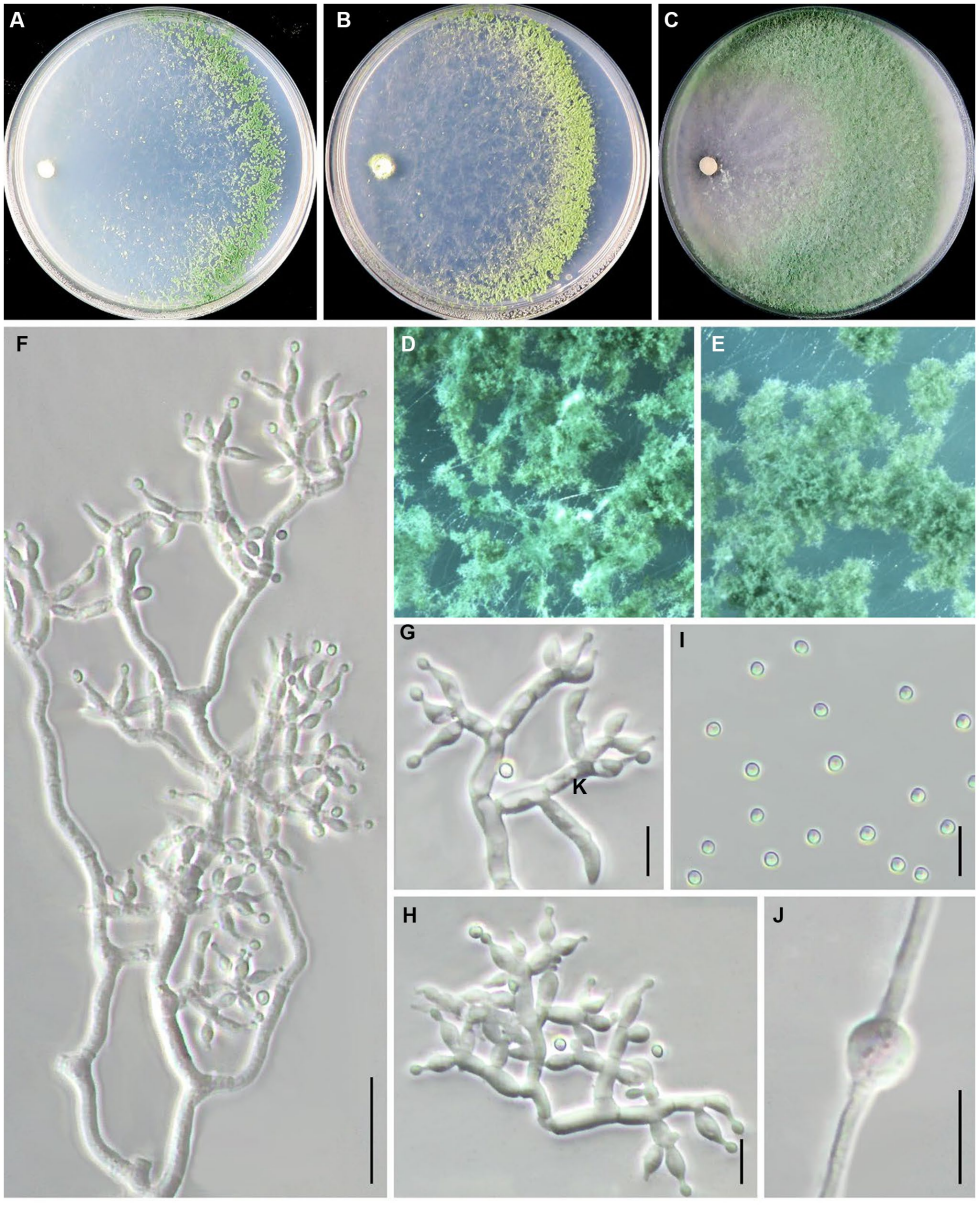
*Trichoderma notatum* (JZBQT1Z5) cultures at 25°C after 7 days [**(A)** on CMD, **(B)** on SNA, **(C)** on PDA]; **(D,E)** conidiation pustules (CMD, 7 days); **(F–H)** conidiophores, phialides, and conidia (SNA, 7 days); **(I)** conidia (SNA, 7 days); **(J)** chlamydospores (PDA, 7 days); scale bars: **(F)** = 20 μm, **(G–J)** = 10 μm.

***Trichoderma notatum*** Z. J. Cao & W.T. Qin, sp. nov. (Figure 5).

MycoBank: MB849038.

*Typification*: China, Beijing, Tongzhou district, 39°41′51” N, 116°45′1″ E, 26 August 2021, W.T. Qin, Y. Liu, S.X. Wang, (ex-type strain JZBQT1Z5). GenBank accessions: *rpb*2 = OP832381, *tef1-α* = OP832396.

*Etymology*: The specific epithet refers to special sequence distinct from other species.

*Description*: On CMD after 72 h, colony radius 60–66 mm at 25°C, covering the plate at 30°C, 34–39 mm at 35°C. Colony hyaline, mycelium loose, more abundant with distance from the original inoculum. Aerial hyphae short, inconspicuous. After 2 days, the margin of the colony formed distinctly zonate, with a large number of white pustules. Conidiation formed on aerial hyphae and pustules, and gradually turn pale green. No diffusing pigment. On PDA after 72 h, colony radius 58–61 mm at 25°C, covering the plate at 30°C, 26–28 mm at 35°C. Colony radial, regularly round. Aerial hyphae white and abundant, flocculent in the middle of the colony. Conidiation effuse in the aerial hyphae after 3 days, first white, then turning green. No diffusing pigment, no distinct odor. On SNA after 72 h, colony radius 53–57 mm at 25°C, 59–62 mm at 30°C, 21–23 mm at 35°C. Colony hyaline, regularly circular, distinctly zonate; mycelium loose. Conidiation noted after 2 days, formed in pustules, first white, turning yellow after 3 days. No diffusing pigment.

Conidiophores pyramidal, with obscure main axis sometimes, often rebranching 2–3 times. The branches are usually at right angles to the main axis, and branch ends are often curved. Phialides formed at the tips of branches most in whorls of 2–4. Phialides ampulliform to lageniform, 4.9–9.9(−11.9) × 2.5–4.3 μm, l/w 1.3–3.3(−3.7), 1.2–2.8 μm wide at the base (*n* = 35). Conidia green, smooth, globose or subglobose, 2.7–3.6 × 2.5–3.1(−3.5) μm, l/w 1.0–1.2 (*n* = 40). Chlamydospores common, intercalary or terminal, most globose or oblong, rare ellipsoid, 4.9–9.6 × 4.1–8.3 μm (*n* = 30).

*Additional strains examined*: China, Beijing, Tongzhou district, 39°41′51” N, 116°45′1″ E, 26 August 2021, W.T. Qin, Y. Liu, S.X. Wang, JZBQT1Z11.

*Notes*: *Trichoderma notatum* was distributed as a separate terminal branch differing from other species. Compared with phylogenetically related species *T. simmonsii*, the phialides of *T. notatum* were longer [(4.2–)5.2–6.5(−9.0) μm], and its growth rate was slower at 35°C [25–55 mm] (Chaverri et al., 2015). The sequence similarity of *rpb2* and *tef1-α* between strain JZBQT1Z5 and *T. simmonsii* (strain S7) was 97.97 and 98.21%, with 23 bp and 22 bp differences among 1,061 bp and 1,231 bp, respectively.

***Trichoderma paratroviride*** Jaklitsch & Voglmayr, Stud. Mycol. 80: 75, 2015. Zhang, Zhang, Chen, Li, Guo & Yang, Shandong Sci. 28: 37. 2015.

*Materials examined*: China, Shanxi Province, Datong, 3 December 2021, Y. Liu, W.T. Qin, JZBQT5Z4, JZBQT5Z6.

*Notes*: *Trichoderma paratroviride* is first discovered on wood and bark of broadleaf trees and shrubs (Jaklitsch and Voglmayr, 2015) and isolated from a monitoring of indoor air in the oak mushroom cultivation houses (Ahn et al., 2018). Furthermore, *T. paratroviride* might have been considered as a competitor to edible fungi in 2012 (Kim et al., 2012). The strain JZBQT5Z4 employed in this study exhibited distinct characteristics from *T. paratroviride* isolated in Spain, which manifested as white colonies on PDA (Jaklitsch and Voglmayr, 2015), in contrast to the grayish green and pale yellow colonies observed in our study.

***Trichoderma paraviridescens*** Jaklitsch, Samuels & Voglmayr, Persoonia 31: 128. 2013.

= *Hypocrea viridescens* Jaklitsch & Samuels, in Jaklitsch, Samuels, Dodd, Lu & Druzhinina, Stud. Mycol. 56: 160. 2006.

*Materials examined*: China, Henan Province, Nanyang, 20 April 2021, Y. Liu, W.T. Qin, JZBQL50; China, Hebei Province, Pingquan, 1 January 2022, Y. Liu, W.T. Qin, JZBQT7Z6, JZBQT7Z8, JZBQT7Z9; *ibid.*, 5 March 2022, Y. Liu, W.T. Qin, JZBQT11Z1.

*Notes*: *Trichoderma paraviridescens* is distributed all over the world. Chinese specimens are often collected from rotten wood, plant rhizosphere soil, plant endogenous, and other substrates (Du, 2019). It was characterized by villous dark red stroma in sexual stage, and radial with colony concentric rings, rapid growth at asexual stage. This study was the first report of *T. paraviridescens* isolated from the CSL.

**Figure 6:**
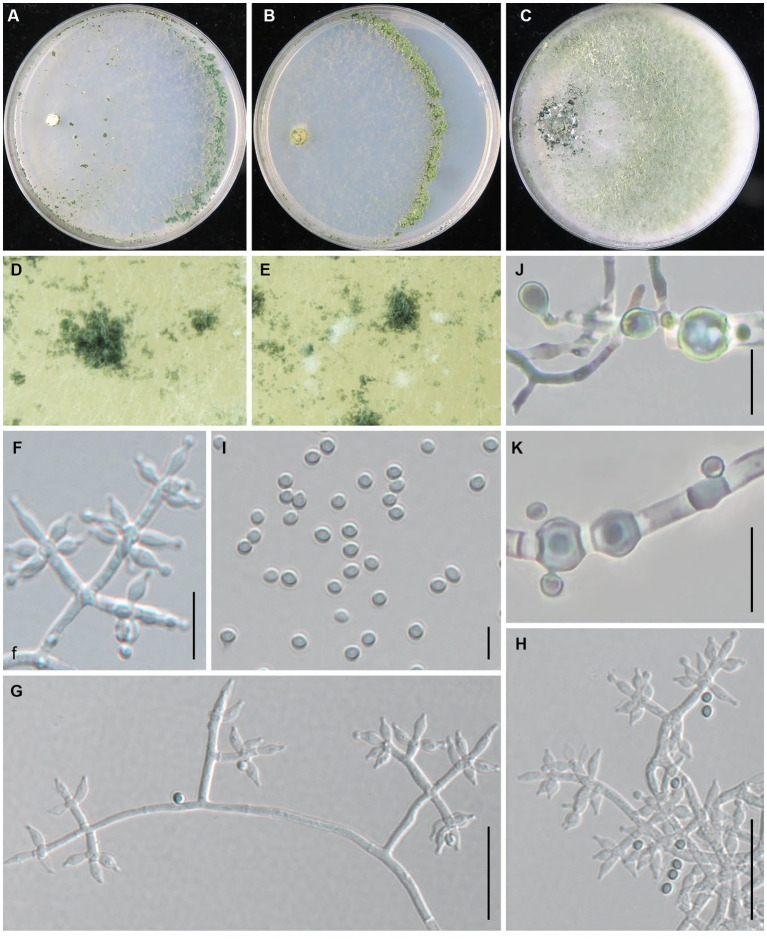
*Trichoderma pingquanense* (JZBQT7Z10) cultures at 25°C after 7 days [**(A)** on CMD, **(B)** on SNA, **(C)** on PDA]; **(D,E)** conidiation pustules (CMD, 7 days); **(F–H)** conidiophores, phialides, and conidia (SNA, 7 days); **(I)** conidia (SNA, 7 days); **(J,K)** chlamydospores (PDA, 7 days); scale bars: **(G,H)** = 20 μm, **(F,J,K)** = 10 μm, **(I)** = 5 μm.

***Trichoderma pingquanense*** Z. J. Cao & W.T. Qin, sp. nov. (Figure 6).

MycoBank: MB849039.

*Type*: China, Hebei Province, Pingquan, 41°2′6” N, 118°44′1″ E, 1 Jan. 2022, Y. Liu, S.X. Wang, W.T. Qin (ex-type strain JZBQT7Z10). GenBank accessions: ITS = ON653401, *rpb2* = ON649961, *tef1-α* = ON649908.

*Etymology*: The specific epithet refers to the type locality.

*Description*: On CMD after 72 h, colony radius 65–69 mm at 25°C, 72–73 mm at 30°C, 13–16 mm at 35°C. Colony hyaline, indistinctly zonate, mycelium loose. Aerial hyphae short, inconspicuous. Small amount of conidial production noted after 4 days, turning blue-green. Pustules are noted throughout the plate. No diffusing pigment, no distinct odor. On PDA after 72 h, colony radius 62–63 mm at 25°C, 66–72 mm at 30°C, 8–9 mm at 35°C. Colony white to white-green, flocculent, mycelium dense and radial. Aerial hyphae long, conspicuous. Small amount of conidial production noted after 3 days, starting around the original inoculum, effuse in the aerial hyphae. No diffusing pigment, odor fruity. On SNA after 72 h, colony radius 51–52 mm at 25°C, 56–59 mm at 30°C, 5 mm at 35°C. Colony hyaline, regularly circular, distinctly zonate; mycelium loose. Conidial production noted after 4 days, starting around the inoculum, first white, turning dark green, with hairs protruding beyond the surface. No diffusing pigment, no distinct odor.

Conidiophores pyramidal, with a relatively obvious main axis, often rebranching 1–3 times. Side branches solitary or paired, in acute or straight angles with main axis, typically formed in a cruciate whorl of 3–5 phialides. Phialides ampulliform to lageniform, 4.3–8.0 × 2.2–3.1(−3.5) μm, l/w 1.6–3.5, 1.3–1.9 μm wide at the base (*n* = 50). Conidia green, smooth, globose or subglobose, 2.4–3.5 × 2.1–3.0 μm, l/w 1.0–1.2 (*n* = 50). Chlamydospores common, intercalary or terminal, variable in shape, ellipsoid, globose or oblong, (4.0–)4.7–8.4 × 3.9–7.5 μm (*n* = 30).

*Additional strains examined*: China, Hebei Province, Pingquan, 41°2′6” N, 118°44′1″ E, 1 January 2022, Y. Liu, S.X. Wang, W.T. Qin, JZBQT7Z11, JZBQT7Z12.

*Notes*: Phylogenetically, *Trichoderma pingquanense* was sister of *T. tongzhouense*, and they are similar in *tef1-α* sequence, while the similarity of *rpb2* was 97.14% with 32 bp differences among 1,117 bp. It differed from *T. xixiacum* by conspicuous chlamydospores. Compared with *T*. *tongzhouense* and *T*. *auriculariae*, *T*. *pingquanense* had narrower phialides and base of phialides [*T*. *tongzhouense* phialides 2.4–4.3 μm, base of phialides 1.7–2.9 μm, *T*. *auriculariae* phialides 2.7–3.8 μm, base of phialides 1.4–2.7 μm].

**Figure 7:**
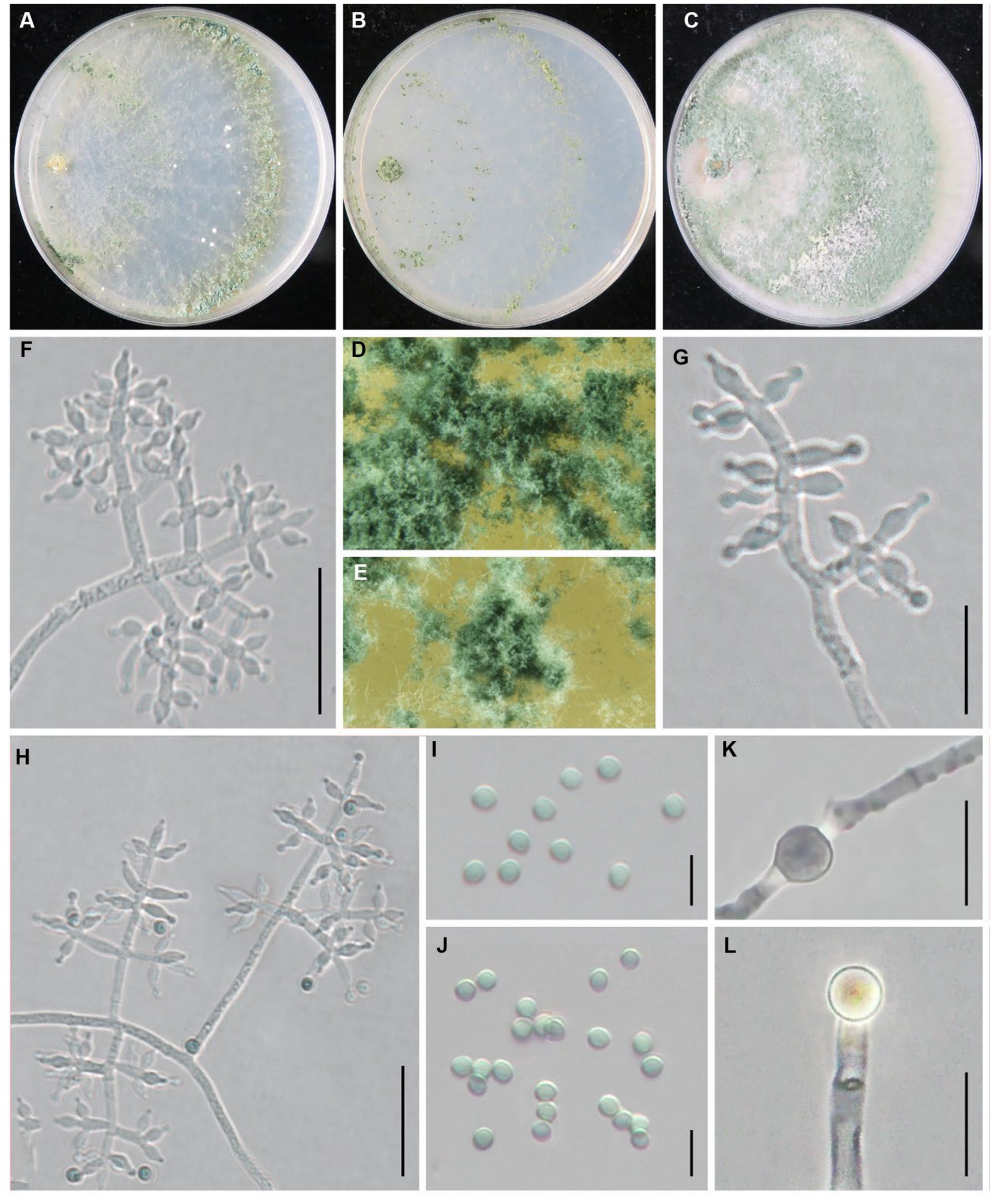
*Trichoderma subvermifimicola* (JZBQT4Z1) cultures at 25°C after 7 days [**(A)** on CMD, **(B)** on SNA, **(C)** on PDA]; **(D,E)** conidiation pustules (CMD, 7 days); **(F–H)** conidiophores, phialides, and conidia (SNA, 7 days); **(I,J)** conidia (SNA, 7 days); **(K,L)** chlamydospores (PDA, 7 days); scale bars: **(F,H)** = 20 μm, **(G,K,L)** = 10 μm, **(I,J)** = 5 μm.

***Trichoderma subvermifimicola*** Z. J. Cao & W.T. Qin, sp. nov. (Figure 7).

MycoBank: MB849041.

*Type*: China, Hebei Province, Baoding, Fuping, 38°53′13” N, 114°0′41″ E, 28 November 2021, Y. Liu, W.T. Qin (ex-type strain JZBQT4Z1). GenBank accessions: ITS = ON653398, *rpb*2 = ON649952, *tef1-α* = ON649899.

*Etymology*: The specific epithet refers to the similarity of the fungus to *T*. *vermifimicola*.

*Description*: On CMD after 72 h, colony radius 63–66 mm at 25°C, 68–70 mm at 30°C, and 14–16 mm at 35°C. Colony hyaline, regularly circular, radial. Conidial production noted after 3 days, scant, effuse in aerial hyphae, concentric circles around the inoculum can be noticed. Sometimes diffusing yellow pigment, no distinct odor. On PDA after 72 h, colony radius 60–64 mm at 25°C, 63–70 mm at 30°C, 13–17 mm at 35°C. Colony white, zonate, mycelium dense and radial. Aerial hyphae long, abundant, spreading throughout the colony, forming a loose, floccose mat. Conidiation noted after 3 days, starting around the original inoculum, effuse in the aerial hyphae. Sometimes diffusing yellow pigment, odor fruity. On SNA after 72 h, colony radius 47–53 mm at 25°C, 54–55 mm at 30°C, 5–7 mm at 35°C. Colony hyaline, indistinctly zonate, mycelium loose. Aerial hyphae loose. Conidiation noted after 3 days, forming conspicuous concentric circles. No diffusing pigment, no distinct odor.

Conidiophores typically pyramidal, symmetry, often with a main axis. Side branches in straight angles with main axis, solitary or paired, rebranching 1–3 times. Branches are perpendicular to the main axis. Phialides formed solitary or paired, and often formed in cruciate whorls of 3–4 at the end of the branch, ampulliform to lageniform, often constricted below the tip to form a narrow neck, (4.2–)4.7–9.4 × 2.3–3.6 μm, l/w 1.4–3.9, (1.2–)1.4–2.1 μm wide at the base (*n* = 50). Conidia green, smooth, globose, sometimes subglobose, 2.7–3.3 × 2.5–3.0 μm, l/w 1.0–1.2(−1.3) (*n* = 50). Chlamydospores common, intercalary or terminal, ellipsoid, globose, 4.0–6.9 × 3.5–6.4 μm (*n* = 30).

*Additional strains examined*: China, Hebei Province, Baoding, Fuping, 38°53′13” N, 114°0′41″ E, 28 November 2021, Y. Liu, W.T. Qin, JZBQT4Z2, JZBQT4Z3, JZBQT4Z4, JZBQT4Z6; China, Hebei Province, Pingquan, 41°2′6” N, 118°44′1″ E, 11 Feb. 2022, W.T. Qin, X.Q. Wang, JZBQT10Z14; *ibid.*, 5 Mar. 2022, Y. Liu, W.T. Qin, JZBQT11Z2, JZBQT11Z3, JZBQT11Z4.

*Notes*: *Trichoderma subvermifimicola* is characterized by typically pyramidal and symmetry conidiophores. Phylogenetically, *T*. *subvermifimicola* is closely associated with *T*. *vermifimicola*. However, they possess 23 bp sequence divergency among 1,206 bp (98.09%) for *rpb2*. *Trichoderma subvermifimicola* exclusively forms obvious concentric circles on CMD and differs from *T. vermifimicola* by larger conidia [(2.0–)2.3–2.6(−3.0) × (1.5–)2.0–2.4(−2.8) μm], and more chlamydospores observed (Gu et al., 2020).

***Trichoderma subviride*** W.T. Qin & W.Y. Zhuang, Scientific Reports 6(27074): 11. 2016.

*Material examined*: China, Hebei Province, Pingquan, 8 January 2022, W.T. Qin, X.Q. Wang, S.X. Wang, JZBQT8Z10, JZBQT8Z11, JZBQT8Z12.

*Notes*: *Trichoderma subviride* was first found on twigs in Henan, China (Qin and Zhuang, 2016), and this is the first report of *T. subviride* isolated from *L. edodes* substrates. *Trichoderma subviride* was featured by pale yellow to yellow green conidiation zones with a squamose surface on PDA (Qin and Zhuang, 2016). Although only three strains among 90 strains investigated were recognized as *T. subviride*, it was still a potential threat to the production of *L. edodes*.

**Figure 8:**
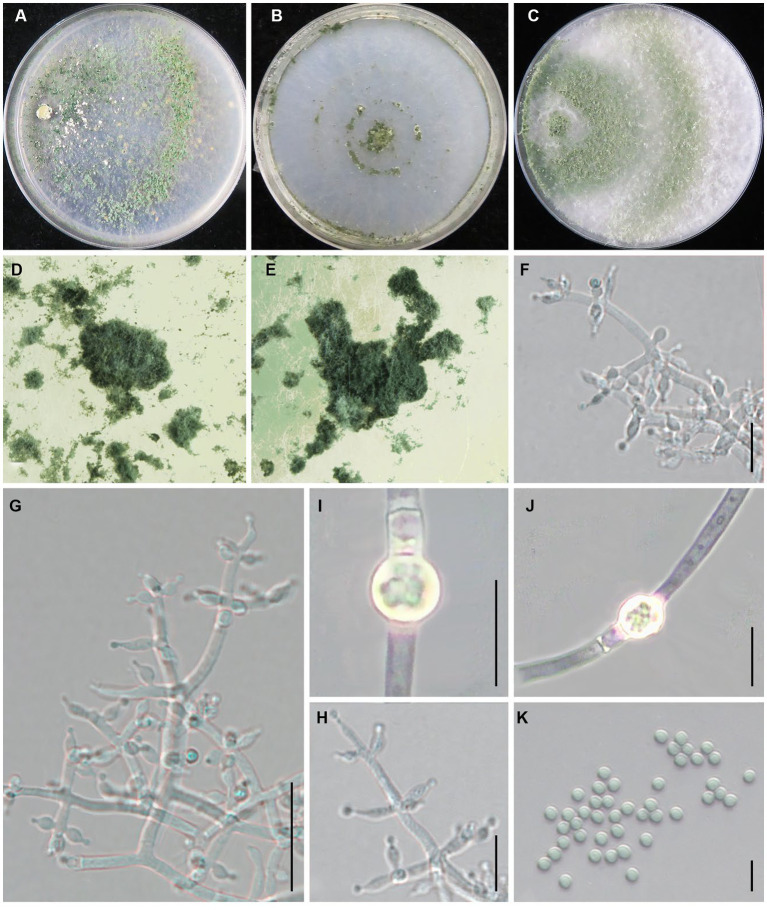
*Trichoderma tongzhouense* (JZBQT1Z1) cultures at 25°C after 7 days [**(A)** on CMD, **(B)** on SNA, **(C)** on PDA]; **(D,E)** conidiation pustules (CMD, 7 days); **(F–H)** conidiophores, phialides, and conidia (SNA, 7 days); **(I,J)** chlamydospores (PDA, 7 days); **(K)** conidia (SNA, 7 days); scale bars: **(G)** = 20 μm, **(F,H)** = 10 μm, **(I–K)** = 5 μm.

***Trichoderma tongzhouense*** Z. J. Cao & W.T. Qin, sp. nov. (Figure 8).

MycoBank: MB849042.

*Type*: China, Beijing, Tongzhou district, 39°41′51” N, 116°45′1″ E, 26 August 2021, W.T. Qin, Z.J. Cao, L. Gao, J. Li (ex-type strain JZBQT1Z1). GenBank accessions: ITS = ON653394, *rpb*2 = ON649945, *tef1-α* = ON649892.

*Etymology*: The specific epithet refers to the type locality.

*Description*: On CMD after 72 h, colony radius 62–70 mm at 25°C, 63–68 mm at 30°C, and 17–20 mm at 35°C. Colony hyaline, circular, weak, mycelium loose. Aerial hyphae inconspicuous, and it was abundant at 30°C. Distinct yellow wheel-like areas appeared at 35°C. Conidia began to appear after 4 days, forming indistinct concentric circles at first, then small pustules spreaded over the entire plate. No diffusing pigment noted, no distinct odor. On PDA after 72 h, colony radius 59–63 mm at 25°C and 62–65 mm 30°C, 13–17 at 35°C. Mycelium white, dense and radial. Aerial hyphae abundant, spreading throughout the colony, forming a loose, floccose mat. Conidia effuse in the aerial hyphae after 3–4 days. No diffusing pigment noted, odor fruity. On SNA after 72 h, colony radius 50–52 mm at 25°C, 54–57 mm at 30°C, 13–15 at 35°C. Colony similar to CMD, mycelium hyaline and smooth, less aerial hyphae. Conidia appeared after 4 days, starting around the inoculum, then turn to green pustules, with the formation of 2–3 concentric rings. No diffusing pigment, no distinct odor.

Conidiophores pyramidal, typically asymmetry, irregularly branched, rebranching 1–3 times. Phialides mostly symmetrically arranged, solitary or divergent in whorls of 2–4(−6), variable in shape and size, ampulliform to lageniform, straight or slightly curved or sinuous, 4.7–13.1(−16.0) × 2.4–4.3 μm, l/w 1.4–4.4(−4.8), 1.7–2.9 μm wide at the base (*n* = 50). Conidia green, globose or subglobose, sometimes ellipsoidal, smooth, 2.7–3.5(−3.8) × 2.4–3.5 μm, l/w 1.0–1.2(−1.3) (*n* = 50). After 10 days, chlamydospores were readily visible at PDA, terminal or intercalary, mostly globose or subglobose, ellipsoidal or fusoid, 5.3–9.2 × 5.2–8.7 μm (*n* = 40).

*Additional strains examined*: China, Beijing, Tongzhou district, 39°41′51” N, 116°45′1″ E, 26 August 2021, W.T. Qin, Z.J. Cao, L. Gao, J. Li, JZBQT1Z2, JZBQT1Z3, JZBQT1Z4.

*Notes*: Phylogenetically, *T*. *tongzhouense* is sister of *T. pingquanense* in the Harzianum Clade. *Trichoderma tongzhouense* possess 32 bp among 1,117 bp *rpb2* sequence difference (97.14%) from *T. pingquanense* (strain JZBQT7Z10). Morphologically, *T*. *tongzhouense* is similar to *T*. *pingquanense* in characteristics of colonies and microscopic morphologies, but the phialide base of *T*. *tongzhouense* is wider than that of *T. pingquanense* [1.3–1.9 μm]. Differs from *T*. *xixiacum* by longer phialides [3.5–7.0 μm] and larger conidia [2.3–2.7 × 2.0–2.6 μm].

### 3.4 Resistance evaluation of *L. edodes* to *Trichoderma* in dual culture

The resistance of *L. edodes* to two *Trichoderma* species was tested in dual culture. The results showed that both *T. macrochlamydospora* and *T*. *subvermifimicola* showed strong inhibition on *L. edodes.* The inhibitory rates of *T. subvermifimicola* against *L. edodes* ranged from 47.04% (*L. edodes* strain SX8) to 58.36% (*L. edodes* strain LHT2). Significance analysis showed that *T. macrochlamydospora* inhibited slightly on *L. edodes* strain SX8 and strain SX10 (Supplementary Figure S2). The inhibitory rates for *T. macrochlamydospora* ranged from 43.27% (*L. edodes* strain SX8) to 58.41% (*L. edodes* strain LHT2). Significance analysis showed that *T. macrochlamydospora* inhibited slightly on *L. edodes* strain SX8 and strain K21 × Z9 (Supplementary Figure S3).

**Figure 9:**
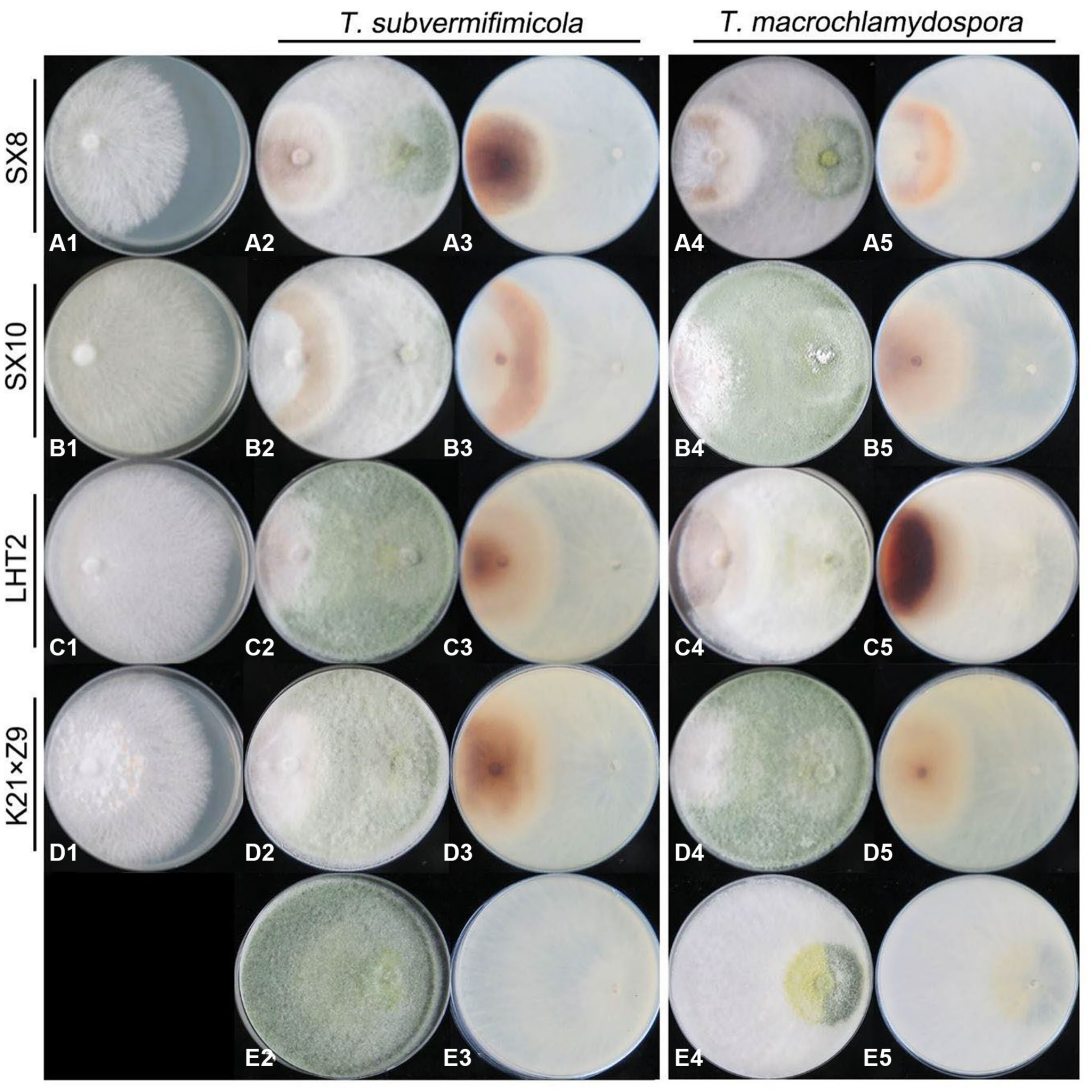
Antagonistic ability of *L. edodes* (left) against *Trichoderma* strains (right) in dual culture. (A–D) + 1, *L. edodes* strains only; (E) + 2–5, *Trichoderma* strains only; [(A–D) + 2, 4] the upper view of the plate in dual culture; [(A–D) + 3, 5] the reverse view of the plate in dual culture.

**Figure 10:**
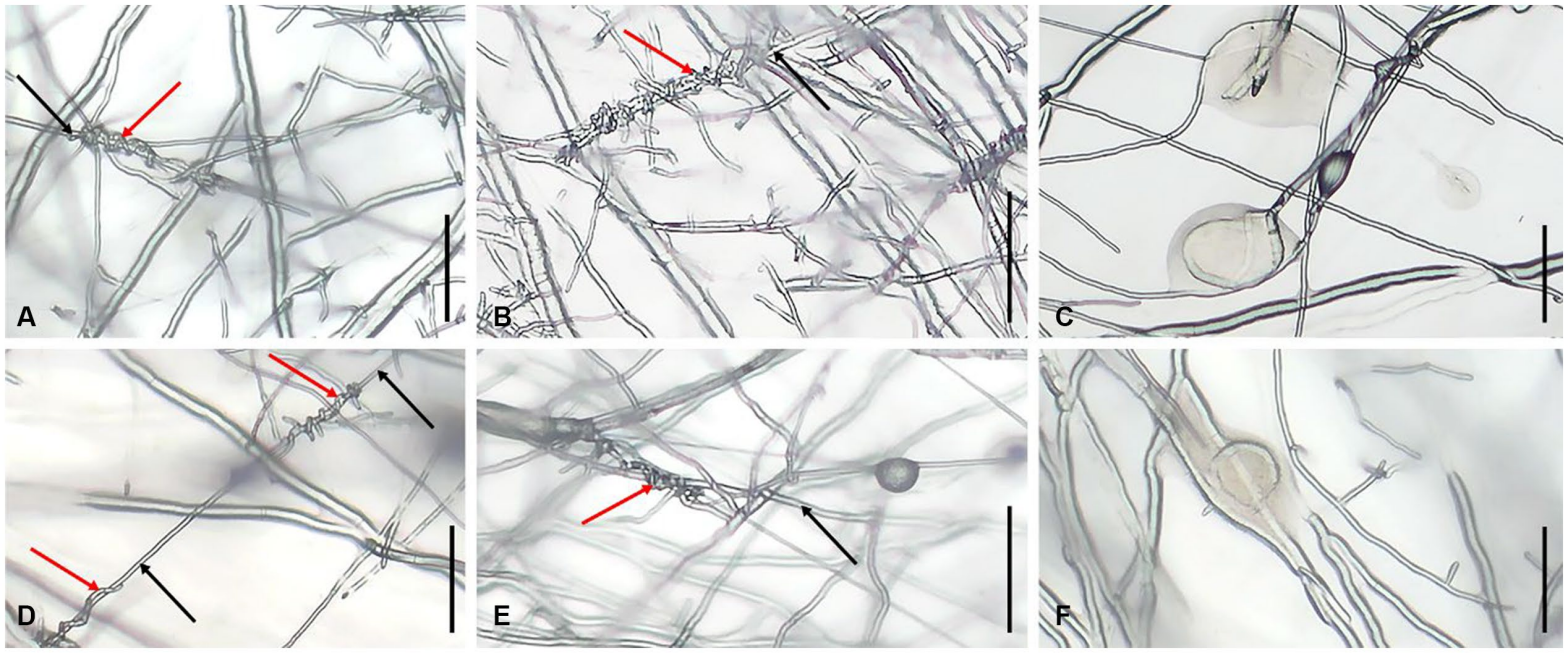
Hyphal state of *L. edodes* contacted with *Trichoderma* spp. **(A)** production of hyphal coilings by *T. subvermifimicola* to *L. edodes* strain SX8; **(B)** production of hyphal coilings by *T. subvermifimicola* to *L. edodes* strain LHT2; **(C)** *L. edodes* strain SN602 exuded brown substance; **(D)** production of hyphal coilings by *T. macrochlamydospora* to *L. edodes* strain SX8; **(E)** production of hyphal coilings by *T. macrochlamydospora* to *L. edodes* strain LHT2; **(F)** *L. edodes* strain XY exuded brown substance; red arrows indicate hyphae of *Trichoderma* spp., and black arrows indicate hyphae of *L. edodes*; scale bars = 100 μm.

*Trichoderma* strains grow approximately faster than *L. edodes*. At the initial stage of hyphae contacted between *Trichoderma* and *L. edodes*, *Trichoderma* spp. strongly inhibited the growth of hyphae of *L. edodes*. *Trichoderma* had also become slow growing, but it was still able to slowly overgrew the territory of *L. edodes* and sporulate abundantly in most plates in 13 days. *Lentinula edodes* would secrete more brown substances on most of the plates, and obvious brown antagonistic lines or entire brown colony of *L. edodes* could be seen on the back of the plates (Figure 9). Simultaneously, both of *Trichoderma* species showed adhesion or coil growth to hyphae of *L. edodes* without significant difference (Figure 10). At times, it was observed that the vesicular structure on the mycelium of *L. edodes* was broken and brown substance flowed out (Figure 10).

## 4 Discussion

Since the artificial cultivation of edible fungi, the serious green mold contamination caused by *Trichoderma* spp. in various growth stage of edible fungi has always been one of the biggest biological constraints in edible fungus industry. Therefore, from 2020 to 2022, edible fungus sticks contaminated with green mold were collected from four provinces of China. The diversity of *Trichoderma* species was relatively abundant, with 90 strains belonging to 14 species. Among them, eight species were located in the clade of Harzianum, two species belonged to the Longibrachiatum clade, and four attributed to the Viride clade. The emergence of green mold contaminated substrates of edible fungi undoubtedly constitutes a prominent hotspot for the diversity of *Trichoderma* species. Six out of the 14 species in the Harzianum clade have been newly established and typified for the first time. Three known *Trichoderma* species, namely, *T. auriculariae*, *T. paraviridescens* and *T. subviride* were isolated from CSL for the first time in the world, and *T. paratroviride* was firstly reported to be associated with *L. edodes* in China. *Trichoderma atroviride*, *T. macrochlamydospora*, and *T. subvermifimicola* were the three most frequently isolated species from the CSL. Consequently, this study lays a foundation for comprehensively understanding *Trichoderma* spp. in the CSL.

Following the gradual refinement and extensive implementation of DNA-based methodologies, the polyphasic taxonomic approach has been advocated for species delimitation (Druzhinina and Kubicek, 2005; Luecking et al., 2020). With the advancement of *Trichoderma* classification research, researchers have such a consensus that the morphological identification of *Trichoderma* with high ambiguity is not suitable for accurate identification of this genus (Chaverri and Samuels, 2003; Jaklitsch et al., 2006). The new species in this study have similar morphological characteristics to some close-related species on the phylogenetic tree. Consequently, phylogenetic analysis became a powerful method to ascertain the placement of ambiguous species, especially for species complexes and cryptic species (Li et al., 2013). According to the species delimitation of Chaverri et al. (2015), most of the new species identified in this study would be considered *Trichoderma harzianum* species complex. In this study, we provided more examples of the strong biocontrol effect of *T. harzianum* complex by showing strong inhibition of *T. subvermifimicola* and *T. macrochlamydospora* in the dual culture with *L. edodes*. In addition, Kim et al. (2012) divided isolated strains into two groups of *T*. *atroviride* by whether formed a dark green ring pustular band in SNA culture. In this study, we found that *T. atroviride* JZBQT7Z1 (JZBQT7Z2, JZBQT7Z3) was more likely to form green rings on PDA than *T. atroviride* JZBQT8Z4 (JZBQT8Z5, JZBQT8Z6), which may also be the result of the accumulation of genetic variations within *T*. *atroviride*. Recent studies and the results in this study enriched the species around *T. lixii* and *T. simmonsii* in the phylogenetic tree, while the relationships between members were more sophisticated, needing further research (Gu et al., 2020).

*Trichoderma* spp. play critical roles in biological control of plant diseases through antagonism against plant pathogens. *Trichoderma* spp. inhabitated in soil, rhizosphere or internal tissues of plant have a variety of mechanisms to protect plants from diseases. One of the most common mechanisms is competition for space and nutrients. In this study, the resistance of *L. edodes* to *Trichoderma* spp. was evaluated, and the results indicated that some *Trichoderma* species also are able to coil around and suck the hyphae of *L. edodes* to make it swell, deform, or even dissolute. They parasitize competing fungi through the production of volatile organic compounds, lytic enzymes, etc., and also can stimulate the defense mechanisms of plant (Nuangmek et al., 2021; Rajani et al., 2021). Meanwhile, volatile and fermented metabolites of *Trichoderma* species also have significant inhibitory effect on the growth of mycelia and fruiting bodies of *L. edodes* (Bruce et al., 1984; Wu et al., 2007). For example, most *Trichoderma* species can produce secondary metabolites and degrading enzymes that can dissolve host cell walls. Nonetheless, the interaction between edible fungi and pathogenic fungi is still a very complex process (Ma et al., 2021). In this study, the potential of *L. edodes* varieties with strong resistance to the dominant *Trichoderma* species on contaminated substrates was screen out. The *L. edodes* strains showed varying degrees of resistance to the two new *Trichoderma* species and *L. edodes* strain SX8 relatively higher resistant. The inhibitory rates of *Trichoderma* against *L. edodes* may be influenced by the growth rate of *L. edodes* mycelium, which should be mentioned. In addition, compared with other edible fungi such as oyster mushroom, almost all varieties of *L. edodes* generally have poor resistance to *Trichoderma* spp. (Wu et al., 2007), which were consistent with our results. Unfortunately, no significant morphology characteristics were found to be related to the infection ability of *Trichoderma* or the resistance of *L. edodes. Trichoderma* sometimes produced fewer green conidia, such as in dual culture of *T. subvermifimicola* against *L. edodes* strain SX10 (Figure 9B2). Moreover, the brown substances secreted by *L. edodes* were less observed on the back of some plates, such as in dual culture of *T. macrochlamydospora* and *L. edodes* strain K21 × Z9 (Figure 9D5). Such differences can exist between different repeats of the same dual culture. However, this phenomenon is difficult to explain in previous studies, which showed that strong resistance of *Trichoderma* to plant pathogenic fungi was associated with the more abundant sporulation of *Trichoderma* (Zhang and Zhuang, 2017). The presence of these uncertainties suggests that the inhibitory effect of *Trichoderma* on *L. edodes* might not be directly linked to sporulation, and the conidium count of *Trichoderma* does not seem to have a direct correlation with the brown substance secreted by *L. edodes*.

This also makes it difficult to distinguish the differences between *Trichoderma* and different *L. edodes* varieties through inhibitory rate, brown substance secreted by *L. edodes* and sporulation by *Trichoderma* in this study. The investigation of various *Trichoderma* spp. on the resistance mechanism of edible fungi will contribute to the research on the resistance of edible fungi against *Trichoderma* (Ma et al., 2019). Considerably, extensive efforts will need to be made to screen and culture-resistant strains of *L. edodes* against *Trichoderma* by breeding techniques. Presently, to minimize *Trichoderma* spp. contamination, it is proposed to examine the *Trichoderma* occurrence mechanism, including its correlation with mushroom substrate and growing environment. Furthermore, the development of an early monitoring system for the predominant *Trichoderma* species is crucial, along with the refinement of cultivation techniques and the identification of highly efficient, low-toxic fungicides.

Remarkably green mold contaminated substrates of edible fungi were undoubtedly a hotspot of *Trichoderma* species diversity. Until now, there are 31 *Trichoderma* species have been reported to be associated with the CSL (Supplementary Table S4). However, our knowledge of the group in the substrates of edible fungi is far from sufficient compared with other origins, such as rotten wood (Zhu and Zhuang, 2015; Qin and Zhuang, 2016) and soil (Chen and Zhuang, 2017). Comprehensive surveys in unexplored territories or alternative edible fungus hosts are undoubtedly necessary.

## 5 Conclusion

To assess the biodiversity of *Trichoderma* in the CSL in China, 150 samples were collected from four provinces of China during a two-year investigation. A total of 90 isolates were obtained and identified as 14 *Trichoderma* species, and their phylogenetic positions were determined, among which six *Trichoderma* species were recognized as new. Three known species, namely, *T. auriculariae*, *T. paraviridescens* and *T. subviride* were isolated from CSL for the first time in the world, and *T. paratroviride* was firstly reported to be associated with *L. edodes* in China. Furthermore, this study systematically investigated *Trichoderma* species in the CSL, and a total of 31 species so far have been reported, indicating that green mold contaminated substrates of edible fungi were undoubtedly a biodiversity hotspot of *Trichoderma* species. In addition, resistance of *L. edodes* to the dominant *Trichoderma* species was evaluated *in vitro*, and the results showed that strains of *L. edodes* generally showed poor resistance to *Trichoderma* contamination with *L. edodes* strain SX8 relatively higher resistant. Results in this study will provide deeper insight into the genus *Trichoderma* and lay a strong foundation for scientific management of the *Trichoderma* contamination in the production of *L. edodes*.

## Data availability statement

The data presented in the study are deposited in Mycobank repository (https://www.mycobank.org/), accession number MB849036, MB849037, MB849038, MB849039, MB849041, MB849042.

## Author contributions

Z-JC: Data curation, Software, Visualization, Writing – original draft. JZ: Formal analysis, Writing – review & editing. YL: Project administration, Investigation. S-XW: Resources. S-YZ: Supervision. W-TQ: Funding acquisition, Conceptualization, Supervision, Writing – review & editing.
