# Supplementary material for: Diversity of Trichoderma species associated with green mold contaminating substrates of Lentinula edodes and their interaction
Source: Front Microbiol. 2024 Jan 8;14:1288585. doi: 10.3389/fmicb.2023.1288585 (PMC10800798; doi:10.3389/fmicb.2023.1288585)
Supplement: Supplementary file 1 [file Data_Sheet_1.pdf]

Diversity of *Trichoderma* species associated with green mold contaminating substrates of *Lentinula edodes* and their interaction

Zi-Jian Cao[1,2], Juan Zhao[1], Yu Liu[1], Shou-Xian Wang[1] , Su-Yue Zheng[2] and Wen-Tao Qin[1]*

[1]Institute of Plant Protection, Beijing Academy of Agriculture and Forestry Sciences, Beijing, China,

[2]School of Landscape and Ecological Engineering, Hebei University of Engineering, Handan, China

Supplementary Material

1 Supplementary Figures and Tables

1.1 Supplementary Figures

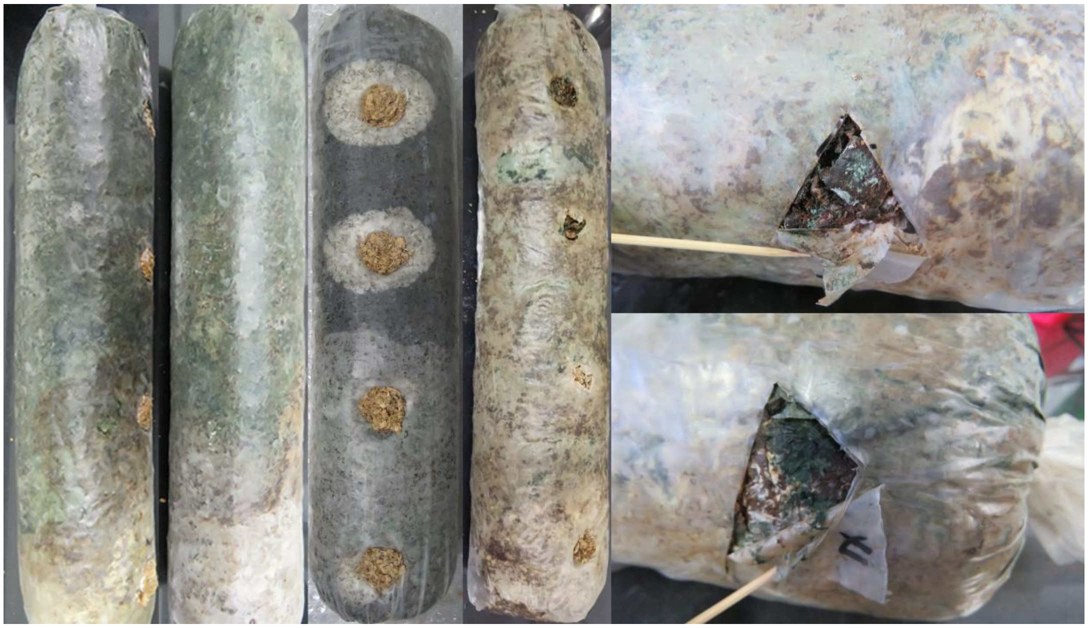

Supplementary Figure S1. The contaminated symptoms of substrates of *Lentinula edodes* contaminated by green mold.

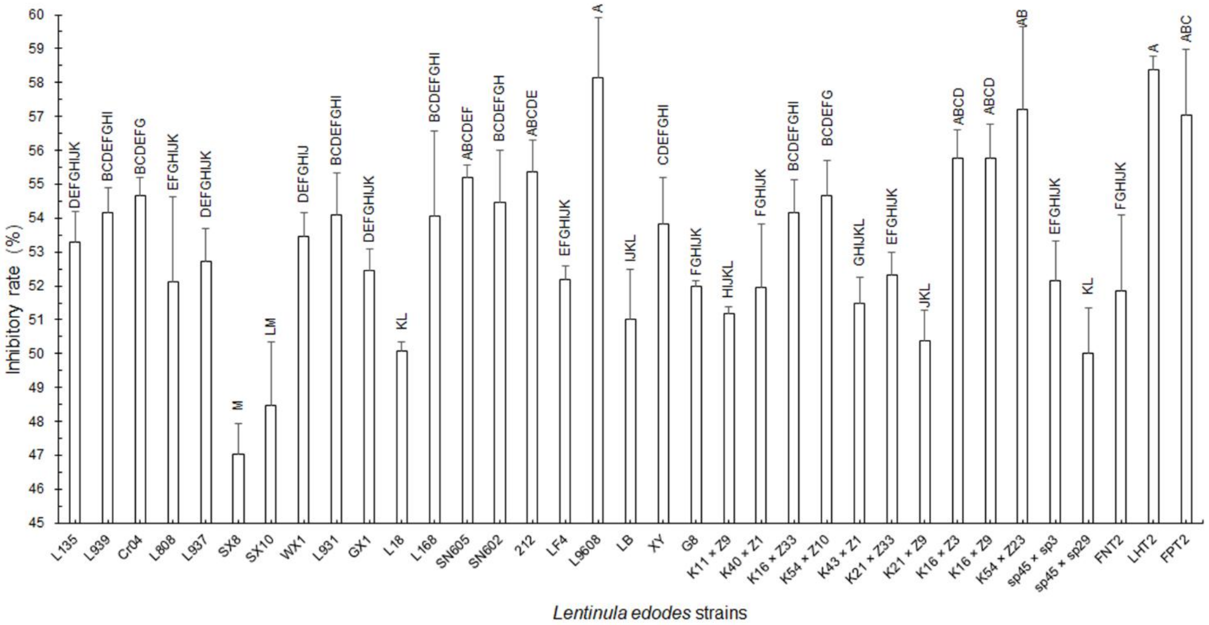

Supplementary Figure S2. Inhibitory rates of *L. edodes* against *T. subvermifimicola.* Different capital letters indicated significant difference at *P*<0.01 level by Duncan's multiple range test.

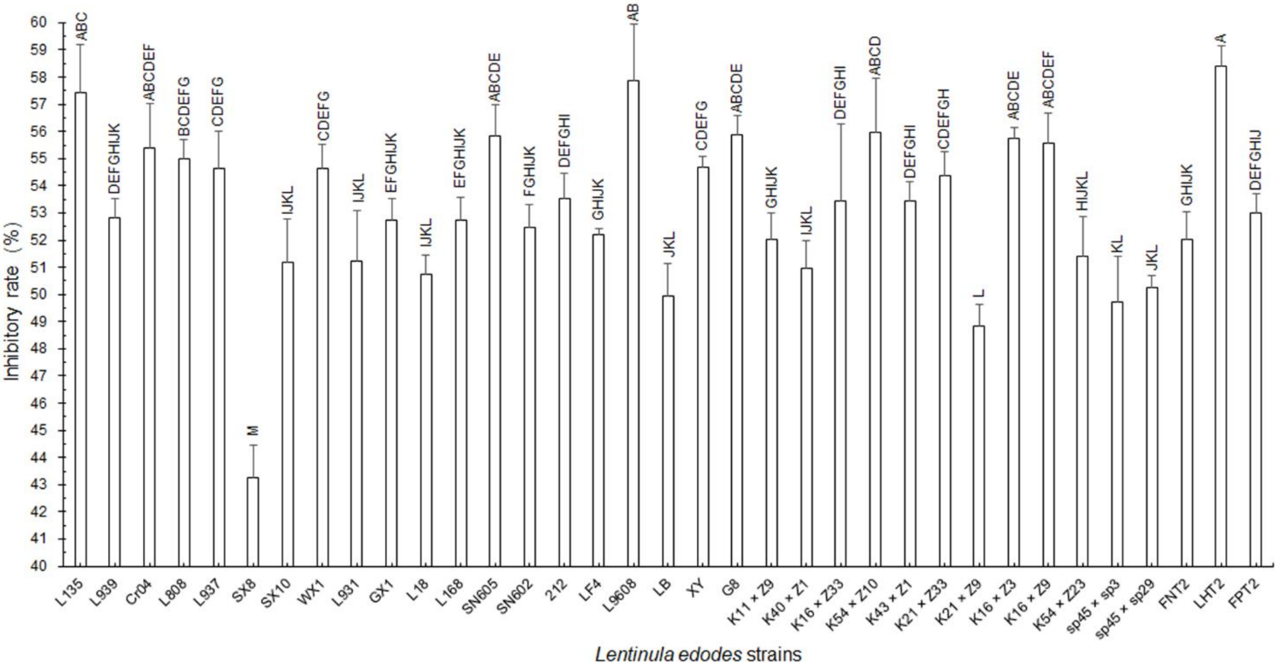

Supplementary Figure S3. Inhibitory rates of *L. edodes* against *T. macrochlamydospora.* Different capital letters indicated significant difference at *P*<0.01 level by Duncan's multiple range test.

1.2 Supplementary Table

Supplementary Table S1. Information including strain numbers and GenBank accession numbers of *Trichoderma* species used for phylogenetic analyses.

Species	Voucher	GenBank Accession Number	
		rpb2	tef1-α
Trichoderma achlamydosporum	YMF 1.6177	MT052180	MT070156
T. adaptatum	HMAS 248800	KX428042	KX428024
T. adaptatum	HMAS 248801	KX428045	KX428027
T. aethiopicum	C.P .K. 1837 T	HM182986	–
T. afarasin	CBS 130755	–	AF348093
T. afarasin	DIS 314F	FJ442778	FJ463400
T. afroharzianum	CBS 124620 ET	FJ442691	FJ463301
T. afroharzianum	GJS 04-193	FJ442709	FJ463298
T. aggregatum	HMAS 248863	KY688001	KY688062
T. aggregatum	HMAS 248864	KY688002	KY688063
T. aggressivum	CBS 100525	AF545541	AF348095
T. aggressivum	DAOM222156 ET	FJ442752	AF348098
T. alni	CBS 120633 ET	EU498349	EU498312
T. alpinum	HMAS 248821 T	KY687958	KY688012
T. amazonicum	IB95	HM142368	HM142377
T. amazonicum	CBS 126898 ET	HM142367	HM142376
T. anaharzianum	YMF 1.00241	MH262577	MH236493
T. anaharzianum	YMF 1.00383 T	MH158995	MH183182
T. andinense	G.J.S. 90-140 T	JN175531	AY956321
T. asiaticum	YMF 1.00168	MH262575	MH236492
T. asiaticum	YMF 1.00352 T	MH158994	MH183183
T. asterineum	HMAS 271353	KT224469	KT224465
T. atrobrunneum	GJS 90-254	FJ442735	AF443943
T. atrobrunneum	GJS 05-101	FJ442745	FJ463392
T. atrogelatinosum	CBS 237.63 ET	KJ842201	–
T. atroviride	CBS 119499	FJ860518	FJ860611
T. atroviride	JZBQT7Z1	ON649980	ON649927
T. atroviride	JZBQT7Z2	ON649981	ON649928
T. atroviride	JZBQT7Z3	ON649982	ON649929
T. atroviride	JZBQT7Z4	/	/
T. atroviride	JZBQT7Z5	/	/
T. atroviride	JZBQT8Z4	ON649983	ON649930
T. atroviride	JZBQT8Z5	ON649984	ON649931
T. atroviride	JZBQT8Z6	ON649985	ON649932
T. atroviride	JZBQT10Z9	ON649989	ON649936
T. atroviride	JZBQT10Z13	ON649990	ON649937
T. atroviride	JZBQT12Z1	ON649991	ON649938
T. atroviride	JZBQT12Z2	ON649992	ON649939
T. atroviride	JZBQT12Z3	ON649993	ON649940
T. atroviride	JZBQT12Z4	/	/

T. auriculariae	JZBQT1Z7 [T]	ON649949	ON649896
T. auriculariae	JZBQT1Z8	ON649950	ON649897
T. auriculariae	JZBQT1Z9	ON649951	ON649898
T. auriculariae	JZBQT10Z10	OP832391	OP832406
T. auriculariae	JZBQT10Z11	OP832392	OP832407
T. austroindianum	BAFC 3583	–	MH352421
T. azevedoi	CEN1422 [T]	MK696821	MK696660
T. bannaense	HMAS 248840 [T]	KY687979	KY688037
T. bannaense	HMAS 248865	KY688003	KY688038
T. beijingense	HMAS 248804 [T]	KX428043	KX428025
T. beijingense	HMAS 248805	KX428044	KX428026
T. bissettii	SFC20170821-M05	–	MN307414
T. botryosum sp.nov.	COAD 2422	MK044212	MK044119
T. botryosum sp.nov.	COAD 2401	MK044181	MK044088
T. breve	HMAS 248844 [T]	KY687983	KY688045
T. breve	HMAS 248845	KY687984	KY688046
T. brunneoviride	CBS 121130 [T]	EU498357	EU498316
T. brunneoviride	CBS 120928	EU498358	EU498318
T. caeruloviride	COAD 2416	MK044201	MK044108
T. caeruloviride	COAD 2415	MK044202	MK044109
T. caesareum	GJS 01-225 [T]	HQ342279	HQ342216
T. caespitosus	JZBQT1Z6 [ET]	OP832383	OP832398
T. caespitosus	JZBQT1Z12	OP832384	OP832399
T. camerunense	GJS 99-230	–	AF348107
T. capillare	GJS 06-66	JN175530	JN175585
T. catoptron	GJS 02-76 [T]	AY391900	AY737726
T. ceraceum	GJS 95-159	AF545508	AY937437
T. ceratophylletum	YMF 1.04621 [T]	MK327580	MK327579
T. cerinum	DAOM230012	KJ842184	KJ871242
T. chlorosporum	GJS 88-33 [T]	AY391903	AY391966
T. christiani	CBS 132572 [ET]	KJ665244	KJ665439
T. cinnamomeum	GJS 96-128	AY391916	AY391977
T. cinnamomeum	GJS 97-233	AY391919	AY391978
T. citrinoviride	S20	KJ665250	KJ665449
T. citrinoviride	JZBQT10Z1	OP832388	OP832403
T. citrinoviride	JZBQT10Z2	OP832389	OP832404
T. citrinoviride	JZBQT10Z3	OP832390	OP832405
T. citrinoviride	JZBQT10Z4	/	/
T. citrinoviride	JZBQT11Z5	/	/
T. citrinoviride	JZBQT11Z6	/	/
T. citrinoviride	JZBQT11Z7	/	/
T. citrinoviride	JZBQT11Z8	/	/
T. compactum	CBS 121218	KF134789	KF134798
T. concentricum	HMAS 248833 [T]	KY687971	KY688027
T. confertum	HMAS 248893	MF371203	MF371218
T. confertum	HMAS 248896	MF371205	MF371220

T. corneum	GJS 97-82 [ET]	KJ665252	KJ665455
T. dacrymycellum	WU29044	FJ860533	FJ860633
T. densum	HMAS 273758	KU529137	KU529126
T. effusum	CPK 254	KJ665260	KJ665473
T. endophyticum	CBS 130753	FJ442722	FJ463326
T. endophyticum	CBS 130733	FJ442690	FJ463330
T. epimyces	CBS 120534 [ET]	EU498360	EU498320
T. euskadiense	S377	KJ665269	KJ665492
T. flagellatum	C.P .K.3345	JN258689	FJ763158
T. gamsii	GJS 04-09	JN133561	DQ307541
T. ganodermatigerum	CCMJ5245 [T]	ON567189	ON567195
T. ganodermatigerum	CCMJ5246	ON567190	ON567196
T. ganodermatigerum	CCMJ5247	ON567191	ON567197
T. globoides	HMAS 248747	KX026963	KX026955
T. gracile	G.J.S. 10-263 [T]	JN175547	JN175598
T. guizhouense	HGUP0038 [T]	JQ901400	JN215484
T. guizhouense	S278	KF134791	KF134799
T. guizhouense	DAOM 231435	–	EF191321
T. hailarense	WT17901 [T]	MH287506	MH287505
T. hailarense	WT17803	MH606232	MH606229
T. harzianum	CBS 226-95	AF545549	AF348101
T. harzianum	GJS 05 107	FJ442708	FJ463329
T. helicolixii	CBS 133499 [T]	KJ665278	KJ665517
T. hengshanicum	HMAS 248852 [T]	KY687991	KY688054
T. hirsutum	HMAS 248834 [T]	KY687972	KY688029
T. hispanicum	S453	JN715600	JN715659
T. hortense	BMCC LU994	–	KJ871185
T. ingratum	HMAS 248822 [T]	KY687973	KY688018
T. inhamatum	CBS 273-78 [T]	FJ442725	AF348099
T. italicum	CBS 132567 [T]	KJ665282	KJ665525
T. jaklitschii	CP61-2 [T]	MW480149	MW480140
T. junci	CBS 120926 [T]	FJ860540	FJ860641
T. koningiopsis	GJS 04-199	FJ442789	FJ463268
T. koreanum	SFC20131005-S066 [T]	MH025988	MH025979
T. kunigamense	TAMA 0193 [T]	AB807657	AB807645
T. laevisporum	HMAS 273756	KU529139	KU529128
T. leguminosarum	S391	KJ665287	KJ665548
T. lentiforme	DIS 253B	FJ442756	FJ851875
T. lentiforme	DIS 94D	FJ442749	FJ463379
T. lentinulae	HMAS 248256 [T]	MN605867	MN605878
T. lentinulae	CGMCC 3.19848	MN605868	MN605879
T. lentinulae	JZBQT0Z1	OP832378	OP832393
T. lentinulae	JZBQT0Z2	OP832379	OP832394
T. lentinulae	JZBQT0Z3	OP832380	OP832395
T. lentinulae	JZBQT0Z4	/	/
T. lentinulae	JZBQT10Z5	/	/

T. lentinulae	JZBQT10Z6	/	/
T. lentinulae	JZBQT10Z7	/	/
T. lentinulae	JZBQT10Z8	/	/
T. liberatum	HMAS 248831 [T]	KY687969	KY688025
T. linzhiense	HMAS 248846 [T]	KY687985	KY688047
T. lixii	CBS 110080 [T]	KJ665290	FJ716622
T. longibrachiatum	CBS 816.68 [T]	DQ087242	EU401591
T. longibrachiatum	S328	JQ685883	JQ685867
T. longifialidicum	LESF 552	KT278955	KT279020
T. longibrachiatum	JZBQT8Z1	ON649994	ON649941
T. longibrachiatum	JZBQT8Z2	ON649995	ON649942
T. longibrachiatum	JZBQT8Z3	/	/
T. longibrachiatum	JZBQT8Z7	ON649996	ON649943
T. longibrachiatum	JZBQT8Z8	ON649997	ON649944
T. longibrachiatum	JZBQT8Z9	/	/
T. longibrachiatum	JZBQL45	/	/
T. longibrachiatum	JZBQL46	/	/
T. martiale	GJS 04-40 [T]	EU248597	EU248618
T. macrochlamydospora	JZBQT5Z1 [ET]	ON649955	ON649902
T. macrochlamydospora	JZBQT5Z2	ON649956	ON649903
T. macrochlamydospora	JZBQT6Z1	ON649957	ON649904
T. macrochlamydospora	JZBQT6Z2	ON649958	ON649905
T. macrochlamydospora	JZBQT6Z3	ON649959	ON649906
T. macrochlamydospora	JZBQT6Z4	ON649960	ON649907
T. macrochlamydospora	JZBQT9Z1	ON649964	ON649911
T. macrochlamydospora	JZBQT9Z2	ON649965	ON649912
T. macrochlamydospora	JZBQT9Z3	ON649966	ON649913
T. macrochlamydospora	JZBQT9Z4	ON649967	ON649914
T. macrochlamydospora	JZBQT9Z5	/	/
T. macrochlamydospora	JZBQT9Z6	/	/
T. macrochlamydospora	JZBQT9Z7	/	/
T. macrochlamydospora	JZBQT9Z8	/	/
T. neokoningii	GJS 04-216 [T]	KJ665318	KJ665620
T. neotropicale	LA11 [ET]	–	HQ022771
T. nordicum	WT13001 [T]	MH287502	MH287501
T. nordicum	WT61001	MH287504	MH287503
T. notatum	JZBQT1Z5 [ET]	OP832381	OP832396
T. notatum	JZBQT1Z11	OP832382	OP832397
T. odoratum	HMAS 271354	KT224468	KT224463
T. orientale	S187	JQ685884	JQ685868
T. parareesei	CBS 125925 [T]	HM182963	GQ354353
T. paratroviride	S385 [T]	KJ665321	KJ665627
T. paratroviride	JZBQT5Z4	ON649978	ON649925
T. paratroviride	JZBQT5Z6	ON649979	ON649926
T. paratroviride	JZBQT10Z12	/	/
T. paraviridescens	Hypo 372	KC285763	DQ672610

T. paraviridescens	JZBQT7Z6	OP832385	OP832400
T. paraviridescens	JZBQT7Z8	/	/
T. paraviridescens	JZBQT7Z9	/	/
T. paraviridescens	JZBQT11Z1	OP832386	OP832401
T. paraviridescens	JZBQL50	OP832387	OP832402
T. parepimyces	CBS 122769 ^{ET}	FJ860562	FJ860664
T. peberdyi	CEN1426 ^{T}	MK696825	MK696664
T. peruvianum	CP15-2 ^{T}	MW480153	MW480145
T. peruvianum	CP15-9	MW480154	MW480146
T. perviride	HMAS 273786 ^{T}	KX026962	KX026954
T. phayaoense	SDBR-CMU349 ^{T}	MW002074	MW002073
T. pingquanense	JZBQT7Z10 ^{ET}	ON649961	ON649908
T. pingquanense	JZBQT7Z11	ON649962	ON649909
T. pingquanense	JZBQT7Z12	ON649963	ON649910
T. pingquanense	JZBQT7Z13	/	/
T. pingquanense	JZBQT7Z14	/	/
T. pingquanense	JZBQT7Z15	/	/
T. pingquanense	JZBQT7Z16	/	/
T. pingquanense	JZBQT7Z17	/	/
T. pinicola	KACC 48486 ^{ET}	MH025993	MH025981
T. pinicola	SFC20130926-S014	MH025991	MH025978
T. pinnatum	G.J.S. 04-100 ^{T}	JN175515	JN175571
T. pleuroti	CBS 124387 ^{ET}	HM142372	HM142382
T. pleuroticola	CBS 124383 ^{ET}	HM142371	HM142381
T. pleuroticola	TRS70 ^{ET}	KP009172	KP008951
T. pluripenicillatum	YMF 1.06198 ^{T}	MT070160	MT070159
T. pollinicola	LC11682 ^{T}	MF939604	MF939619
T. pollinicola	LC11686	MF939605	MF939620
T. polypori	HMAS 248855 ^{T}	KY687994	KY688058
T. priscilae	CBS 131487 ^{ET}	KJ665333	KJ665691
T. propepolypori	YMF 1.06224 ^{T}	MT052181	MT070158
T. propepolypori	YMF 1.06199	MT052182	MT070157
T. pseudoasiaticum	YMF 1.06200 ^{T}	MT052183	MT070155
T. pseudodensum	HMAS 248828 ^{T}	KY687967	KY688023
T. pseudogelatinosum	CNUN309 ^{ET}	HM920173	HM920202
T. pseudopyramidale	COAD 2419	MK044206	MK044113
T. pseudopyramidale	COAD 2506	MK044207	MK044114
T. pubescens	GJS 01-207	FJ150768	EU856304
T. purpureum	HMAS 273787 ^{T}	KX026961	KX026953
T. pyramidale	CBS 135574 ^{ET}	KJ665334	KJ665699
T. reesei	G.J.S. 00-89	JN175548	JN175599
T. rifaii	CBS 130746 ^{T}	–	FJ463324
T. rifaii	DIS 337F	FJ442720	FJ463321
T. rufobrunneum	HMAS 266614 ^{T}	KF730010	KF729989
T. rugulosum	SFC20180301-001 ^{T}	MH025986	MH025984
T. rugulosum	SFC20180301-002	MH025987	MH025985

T. samuelsii	S5	JN715599	JN715655
T. saturnisporum	ATCC 28023	JN175524	JN388897
T. scalesiae	GJS 03-74	EU252007	DQ841726
T. simile	YMF 1.06201 [T]	MT052184	MT070154
T. simile	YMF 1.06202	MT052185	MT070153
T. simmonsii	CBS 130431	FJ442757	AF443935
T. simmonsii	S7	KJ665337	KJ665719
T. simplex	HMAS 248842 [T]	KY687981	KY688041
T. sinuosum	CPK 1595	FJ179619	FJ860697
T. solum	HMAS 248848 [T]	KY687987	KY688050
T. speciosum	YMF 1.00205	MH155270	MH183184
T. sphaerosporum	HMAS 273763	KU529145	KU529134
T. stilbohypoxyli	CBS 119501	FJ860593	FJ860703
T. stramineum	GJS02-84 [T]	AY391945	AY391999
T. stromaticum	GJS 97-183	HQ342245	AY937418
T. subalni	HMAS 275683	MH612371	MH612377
T. subalni	HMAS 275684	MH612370	MH612376
T. subeffusum	CBS 120929 [T]	FJ860597	FJ860707
T. subvermifimicola	JZBQT4Z1 [ET]	ON649952	ON649899
T. subvermifimicola	JZBQT4Z2	ON649953	ON649900
T. subvermifimicola	JZBQT4Z3	ON649954	ON649901
T. subvermifimicola	JZBQT4Z4	/	/
T. subvermifimicola	JZBQT4Z6	/	/
T. subvermifimicola	JZBQT10Z14	/	/
T. subvermifimicola	JZBQT11Z2	/	/
T. subvermifimicola	JZBQT11Z3	/	/
T. subvermifimicola	JZBQT11Z4	/	/
T. subviride	HMAS 273761 [T]	KU529142	KU529131
T. subviride	JZBQT8Z10	ON649986	ON649933
T. subviride	JZBQT8Z11	ON649987	ON649934
T. subviride	JZBQT8Z12	ON649988	ON649935
T. syagri	BAFC 4357	–	MG822711
T. tawa	CBS 114233 [ET]	AY391956	FJ463313
T. tawa	DAOM 232841	KJ842187	EU279972
T. tenue	HMAS 273785 [ET]	KX026960	KX026952
T. tomentosum	DAOM178713a	AF545557	AY750882
T. tongzhouense	JZBQT1Z1 [ET]	ON649945	ON649892
T. tongzhouense	JZBQT1Z2	ON649946	ON649893
T. tongzhouense	JZBQT1Z3	ON649947	ON649894
T. tongzhouense	JZBQT1Z4	ON649948	ON649895
T. tsugarense	TAMA 0203 [T]	AB807659	AB807647
T. valdunense	CBS 120923	FJ860605	FJ860717
T. velutinum	CPK 298	KF134794	KJ665769
T. velutinum	DAOM 230013 [ET]	JN133569	AY937415
T. vermifimicola	CGMCC 3.19850	MN605870	MN605881
T. vermifimicola	HMAS 248255 [T]	MN605871	MN605882

T. vinosum	GJS 99-158	KC285779	AY376047
T. virgineum	HMAS 275664	MG383489	MG383495
T. viridarium	S136	KC285760	KC285658
T. viride	CBS 119325	EU711362	DQ672615
T. viridescens	S452	KC285758	KC285646
T. viridialbum	S250 [T]	KC285774	KC285706
T. xanthum	HMAS 247202	MF371211	MF371226
T. xixiacum	HMAS 248253 [T]	MN605874	MN605885
T. xixiacum	CGMCC 3.19698	MN605875	MN605886
T. zayuense	HMAS248835 [T]	KY687974	KY688031
T. zelobreve	HMAS 248254 [T]	MN605872	MN605883
T. zelobreve	CGMCC 3.19696	MN605873	MN605884
T. zeloharzianum	YMF1.00268 [T]	MH158996	MH183181
Nectria berolinensis	CBS 127382	HM534883	HM534872
N. eustromatica	CBS 121896	HM534886	HM534875

Numbers in bold indicate newly submitted sequences in this study. [T]: type strains. [ET]: ex-type strains. –: unacquired sequence. /: sequences that have been sequenced but not uploaded in this study.

Supplementary Table S2 The information of the tested strains of *L. edodes*.

**Number**	**Strain**	**Number**	**Strain**	**Number**	**Strain**
1	L135	13	SN605	25	K43 × Z1
2	L939	14	SN602	26	K21 × Z33
3	Cr04	15	212	27	K21 × Z9
4	L808	16	LF4	28	K16 × Z3
5	L937	17	L9608	29	K16 × Z9
6	SX8	18	LB	30	K54 × Z23
7	SX10	19	XY	31	sp45 × sp3
8	WX1	20	G8	32	sp45 × sp29
9	L931	21	K11 × Z9	33	FNT2
10	GX1	22	K40 × Z1	34	LHT2
11	L18	23	K16 × Z33	35	FPT2
12	L168	24	K54 × Z10		

Supplementary Table S3 Information of *Trichoderma* specimens collected in this study.

Voucher	Species	Source	Collecting date
JZBQT0Z1	*T. lentinulae*	Beijing, Haidian	2021.8.3
JZBQT0Z2	*T. lentinulae*	Beijing, Haidian	2021.8.3
JZBQT0Z3	*T. lentinulae*	Beijing, Haidian	2021.8.3
JZBQT0Z4	*T. lentinulae*	Beijing, Haidian	2021.8.3
JZBQT1Z1	*T. tongzhouense*	Beijing, Tongzhou	2021.8.26
JZBQT1Z2	*T. tongzhouense*	Beijing, Tongzhou	2021.8.26
JZBQT1Z3	*T. tongzhouense*	Beijing, Tongzhou	2021.8.26
JZBQT1Z4	*T. tongzhouense*	Beijing, Tongzhou	2021.8.26
JZBQT1Z5	*T. notatum*	Beijing, Tongzhou	2021.8.26
JZBQT1Z6	*T. caespitosus*	Beijing, Tongzhou	2021.8.26
JZBQT1Z11	*T. notatum*	Beijing, Tongzhou	2021.8.26
JZBQT1Z12	*T. caespitosus*	Beijing, Tongzhou	2021.8.26
JZBQT5Z1	*T. macrochlamydospora*	Shanxi, Datong	2021.12.3
JZBQT5Z2	*T. macrochlamydospora*	Shanxi, Datong	2021.12.3
JZBQT5Z4	*T. paratroviride*	Shanxi, Datong	2021.12.3
JZBQT5Z6	*T. paratroviride*	Shanxi, Datong	2021.12.3
JZBQT4Z1	*T. subvermifimicola*	Hebei, Baoding	2021.11.28
JZBQT4Z2	*T. subvermifimicola*	Hebei, Baoding	2021.11.28
JZBQT4Z3	*T. subvermifimicola*	Hebei, Baoding	2021.11.28
JZBQT4Z4	*T. subvermifimicola*	Hebei, Baoding	2021.11.28
JZBQT4Z6	*T. subvermifimicola*	Hebei, Baoding	2021.11.28
JZBQT6Z1	*T. macrochlamydospora*	Hebei, Chengde	2021.12.19
JZBQT6Z2	*T. macrochlamydospora*	Hebei, Chengde	2021.12.19
JZBQT6Z3	*T. macrochlamydospora*	Hebei, Chengde	2021.12.19
JZBQT6Z4	*T. macrochlamydospora*	Hebei, Chengde	2021.12.19
JZBQT9Z1	*T. macrochlamydospora*	Hebei, Chengde	2022.1.12
JZBQT9Z2	*T. macrochlamydospora*	Hebei, Chengde	2022.1.12
JZBQT9Z3	*T. macrochlamydospora*	Hebei, Chengde	2022.1.12
JZBQT9Z4	*T. macrochlamydospora*	Hebei, Chengde	2022.1.12
JZBQT9Z5	*T. macrochlamydospora*	Hebei, Chengde	2022.1.12
JZBQT9Z6	*T. macrochlamydospora*	Hebei, Chengde	2022.1.12
JZBQT9Z7	*T. macrochlamydospora*	Hebei, Chengde	2022.1.12
JZBQT9Z8	*T. macrochlamydospora*	Hebei, Chengde	2022.1.12
JZBQT7Z1	*T. atroviride*	Hebei, Pingquan	2022.1.1
JZBQT7Z2	*T. atroviride*	Hebei, Pingquan	2022.1.1
JZBQT7Z3	*T. atroviride*	Hebei, Pingquan	2022.1.1
JZBQT7Z4	*T. atroviride*	Hebei, Pingquan	2022.1.1
JZBQT7Z5	*T. atroviride*	Hebei, Pingquan	2022.1.1
JZBQT7Z6	*T. paraviridescens*	Hebei, Pingquan	2022.1.1
JZBQT7Z8	*T. paraviridescens*	Hebei, Pingquan	2022.1.1
JZBQT7Z9	*T. paraviridescens*	Hebei, Pingquan	2022.1.1
JZBQT7Z10	*T. pingquanense*	Hebei, Pingquan	2022.1.1
JZBQT7Z11	*T. pingquanense*	Hebei, Pingquan	2022.1.1
JZBQT7Z12	*T. pingquanense*	Hebei, Pingquan	2022.1.1
JZBQT7Z13	*T. pingquanense*	Hebei, Pingquan	2022.1.1
JZBQT7Z14	*T. pingquanense*	Hebei, Pingquan	2022.1.1
JZBQT7Z15	*T. pingquanense*	Hebei, Pingquan	2022.1.1
JZBQT7Z16	*T. pingquanense*	Hebei, Pingquan	2022.1.1
JZBQT7Z17	*T. pingquanense*	Hebei, Pingquan	2022.1.1
JZBQT8Z1	*T. longibrachiatum*	Hebei, Pingquan	2022.1.8
JZBQT8Z2	*T. longibrachiatum*	Hebei, Pingquan	2022.1.8

JZBQT8Z3	*T. longibrachiatum*	Hebei, Pingquan	2022.1.8
JZBQT8Z4	*T. atroviride*	Hebei, Pingquan	2022.1.8
JZBQT8Z5	*T. atroviride*	Hebei, Pingquan	2022.1.8
JZBQT8Z6	*T. atroviride*	Hebei, Pingquan	2022.1.8
JZBQT8Z7	*T. longibrachiatum*	Hebei, Pingquan	2022.1.8
JZBQT8Z8	*T. longibrachiatum*	Hebei, Pingquan	2022.1.8
JZBQT8Z9	*T. longibrachiatum*	Hebei, Pingquan	2022.1.8
JZBQT8Z10	*T. subviride*	Hebei, Pingquan	2022.1.8
JZBQT8Z11	*T. subviride*	Hebei, Pingquan	2022.1.8
JZBQT8Z12	*T. subviride*	Hebei, Pingquan	2022.1.8
JZBQT10Z1	*T. citrinoviride*	Hebei, Pingquan	2022.2.11
JZBQT10Z2	*T. citrinoviride*	Hebei, Pingquan	2022.2.11
JZBQT10Z3	*T. citrinoviride*	Hebei, Pingquan	2022.2.11
JZBQT10Z4	*T. citrinoviride*	Hebei, Pingquan	2022.2.11
JZBQT10Z5	*T. lentinulae*	Hebei, Pingquan	2022.2.11
JZBQT10Z6	*T. lentinulae*	Hebei, Pingquan	2022.2.11
JZBQT10Z7	*T. lentinulae*	Hebei, Pingquan	2022.2.11
JZBQT10Z8	*T. lentinulae*	Hebei, Pingquan	2022.2.11
JZBQT10Z9	*T. atroviride*	Hebei, Pingquan	2022.2.11
JZBQT10Z10	*T. auriculariae*	Hebei, Pingquan	2022.2.11
JZBQT10Z11	*T. auriculariae*	Hebei, Pingquan	2022.2.11
JZBQT10Z12	*T. paratroviride*	Hebei, Pingquan	2022.2.11
JZBQT10Z13	*T. atroviride*	Hebei, Pingquan	2022.2.11
JZBQT10Z14	*T. subvermifimicola*	Hebei, Pingquan	2022.2.11
JZBQT11Z1	*T. paraviridescens*	Hebei, Pingquan	2022.3.5
JZBQT11Z2	*T. subvermifimicola*	Hebei, Pingquan	2022.3.5
JZBQT11Z3	*T. subvermifimicola*	Hebei, Pingquan	2022.3.5
JZBQT11Z4	*T. subvermifimicola*	Hebei, Pingquan	2022.3.5
JZBQT11Z5	*T. citrinoviride*	Hebei, Pingquan	2022.3.5
JZBQT11Z6	*T. citrinoviride*	Hebei, Pingquan	2022.3.5
JZBQT11Z7	*T. citrinoviride*	Hebei, Pingquan	2022.3.5
JZBQT11Z8	*T. citrinoviride*	Hebei, Pingquan	2022.3.5
JZBQT12Z1	*T. atroviride*	Hebei, Pingquan	2022.3.5
JZBQT12Z2	*T. atroviride*	Hebei, Pingquan	2022.3.5
JZBQT12Z3	*T. atroviride*	Hebei, Pingquan	2022.3.5
JZBQT12Z4	*T. atroviride*	Hebei, Pingquan	2022.3.5
JZBQL45	*T. longibrachiatum*	Henan, Nanyang	2021.4.20
JZBQL46	*T. longibrachiatum*	Henan, Nanyang	2021.4.20
JZBQL50	*T. paraviridescens*	Henan, Nanyang	2021.4.20

Supplementary Table S4 *Trichoderma* spp. reported to be associated with the CSL.

No.	Species	Cultivated Mushroom	Reference
1	*T. atrobrunneum*	*L. edodes*	[1]
2	*T. atroviride**	*L. edodes*, *Pleurotus ostreatus*, *Agaricus bisporus*, *Ganoderma lingzhi*	[2,3,4], this study
3	*T. aureoviride*	*Auricularia heimuer*, *Flammulina filiformis*, *L. edodes*	[5]
4	*T. auriculariae*⁺	*L. edodes*	This study
5	*T. breve*	*L. edodes*	[6]
6	***T. caespitosus***	*L. edodes*	This study
7	*T. citrinviride*	*L. edodes*, *P. ostreatus*	[4,7], this study
8	*T. deliquescens*	*L. edodes*	[8]
9	*T. guizhouense*	*L. edodes*	[1]
10	*T. harzianum*	*L. edodes*, *A. bisporus*, *P. ostreatus*, *Agrocybe aegerita*	[4,9]
11	*T. hirsutum*	*L. edodes*	[6]
12	*T. lentinulae*	*L. edodes*	[10], this study
13	*T. longibrachiatum*	*L. edodes*, *P. ostreatus*, *A. aegerita*	[3,4,9], this study
14	***T. macrochlamydospora****	*L. edodes*	This study
15	***T. notatum***	*L. edodes*	This study
16	*T. oblongisporum*	*L. edodes*	[11]
17	*T. paratroviride*	*L. edodes*	[16], This study
18	*T. paraviridescens*⁺	*L. edodes*	This study
19	***T. pingquanense***	*L. edodes*	This study
20	*T. pleuroticola*	*P. ostreatus*, *L. edodes*, *G. lingzhi*	[9,11,12]
21	*T. pollinicola*	*L. edodes*	[1]
22	*T. polysporum*	*L. edodes*	[13]
23	*T. pseudogelatinosum*	*L. edodes*	[14]
24	*T. pseudolacteum*	*L. edodes*	[15]
25	*T. pseudostramineum*	*L. edodes*	[14]
26	*T. simmonsii*	*L. edodes*	[1]
27	*T. stramineum*	*L. edodes*	[13]
28	***T. subvermifimicola****	*L. edodes*	This study
29	*T. subviride*⁺	*L. edodes*	This study
30	***T. tongzhouense***	*L. edodes*	This study
31	*T. viride*	*L. edodes*	[11]

Bold font indicate new species proposed in this study. *: dominant species in the CSL. ⁺: first reported in the CSL in this study.

References

[1] Henrietta A.; Zhumakayev A.; Buechner R.; Hatvani L. Members of the *Trichoderma harzianum* species complex with mushroom pathogenic potential. AGRONOMY-BASEL 2021, 11, 2434. doi: 10.3390/agronomy11122434

[2] Yan, Y.; Zhang, C.; Moodley, O.; Zhang, L.; Xu, J. Green mold caused by *Trichoderma atroviride* on the lingzhi medicinal mushroom, Ganoderma lingzhi (Agaricomycetes). Int. J. Med. Mushrooms 2019, 21, 515–521. doi: 10.1615/IntJMedMushrooms.2019030352

[3] Hatvani, L.; Antal, Z.; Manczinger, L.; Szekeres, A.; Druzhinina, I.S.; Kubicek, C.P.; Nagy, A.; Nagy, E.; Vagvolgyi, C.; Kredics, L. Green mold diseases of *Agaricus* and *Pleurotus* spp. are caused by related but phylogenetically different *Trichoderma* species. Phytopathology 2007, 97, 532–537. doi: 10.1094/PHYTO-97-4-0532

[4] Kim, C.S.; Park, M.S.; Kim, S.C.; Maekawa, N.; Yu, S.H. Identification of *Trichoderma*, a competitor of shiitake mushroom (*Lentinula edodes*), and competition between *Lentinula edodes* and *Trichoderma* species in Korea. Plant Pathol. J. 2012, 28, 137–148. doi: 10.5423/PPJ.2012.28.2.137

[5] Cui, L.H. Isolation, Identification and diversity analysis of the contaminating fungi from the edible mushroom-growing synthetic wood logs. Master's Thesis, Liaoning Normal University, Dalian, China, 2017. doi:

[6] Wang, Y. Identification of the Pathogen of *Lentinus edodes* sticks rot and preliminary study on its occurrence. Master's Thesis, Guizhou University, Guiyang, China, 2021.

[7] Park, M.S.; Seo, G.S.; Bae, K.S.; Yu, S.H. Characterization of *Trichoderma* spp. associated with green mold of oyster mushroom by PCR-RFLP and sequence analysis of ITS regions of rDNA. Plant Pathol. J. 2005, 21, 229–236. doi:

[8] Kim, J.Y.; Yun, Y.H.; Hyun, M.W.; Kim, M.H.; Kim, S.H. Identification and characterization of *Gliocladium viride* isolated from mushroom fly infested oak log beds used for shiitake cultivation. Mycobiology 2010, 38, 7–12. doi: 10.4489/MYCO.2010.38.1.007

[9] Choi, I.Y.; Choi, J.N.; Hyu, L.W.; Sharma, P.K. Isolation and identification of mushroom pathogens from *Agrocybe aegerita*. Mycobiology 2010, 38, 310–315. doi: 10.4489/MYCO.2010.38.4.310

[10] Gu, X.; Wang, R.; Sun, Q.; Wu, B.; Sun, J.-Z. Four new species of *Trichoderma* in the Harzianum clade from northern China. Mycokeys 2020, 73, 109–132. doi: 10.3897/mycokeys.73.51424

[11] Wang, G.; Cao, X.; Ma, X.; Guo, M.; Liu, C.; Yan, L.; Bian, Y. Diversity and effect of *Trichoderma* spp. associated with green mold disease on *Lentinula edodes* in China. Microbiologyopen 2016, 5, 709–718. doi: 10.1002/mbo3.364

[12] Park, M.S.; Bae, K.S.; Yu, S.H. Two new species of *Trichoderma* associated with green mold of oyster mushroom cultivation in Korea. Mycobiology 2006, 34, 111–113. doi: 10.4489/MYCO.2006.34.3.111

[13] Miyazaki, K.; Tsuchiya, Y.; Okuda, T. Specific PCR assays for the detection of *Trichoderma harzianum* causing green mold disease during mushroom cultivation. Mycoscience 2009, 50, 94–99. doi: 10.1007/s10267-008-0460-2

[14] Kim, C.S.; Yu, S.H.; Nakagiri, A.; Shirouzu, T.; Sotome, K.; Kim, S.C.; Maekawa, N. Re-evaluation of *Hypocrea pseudogelatinosa* and *H. pseudostraminea* isolated from shiitake mushroom (*Lentinula edodes*) cultivation in Korea and Japan. Plant Pathol. J. 2012, 28, 341–356. doi: 10.5423/PPJ.OA.05.2012.0068

[15] Kim, C.S.; Shirouzu, T.; Nakagiri, A.; Sotome, K.; Maekawa, N. *Trichoderma eijii* and *T. pseudolacteum*, two new species from Japan. Mycol. Prog. 2013, 12, 739–753. doi: 10.1007/s11557-012-0886-y

[16] Ahn, G.R., Kim, J.E., Kim, J.Y., Kim, S.H. Unrecorded fungal species isolated from indoor air in the log bed- and sawdust media-based mushroom cultivation houses. Kor. J. Mycol. 2018, 46, 495-503. doi: 10.4489/KJM.2018005
